# A targeted multi-proteomics approach generates a blueprint of the ciliary ubiquitinome

**DOI:** 10.3389/fcell.2023.1113656

**Published:** 2023-01-26

**Authors:** Mariam G. Aslanyan, Cenna Doornbos, Gaurav D. Diwan, Zeinab Anvarian, Tina Beyer, Katrin Junger, Sylvia E. C. van Beersum, Robert B. Russell, Marius Ueffing, Alexander Ludwig, Karsten Boldt, Lotte B. Pedersen, Ronald Roepman

**Affiliations:** ^1^ Department of Human Genetics, Radboud Institute for Molecular Life Sciences, Radboud University Medical Center, Nijmegen, Netherlands; ^2^ BioQuant, Heidelberg University, Heidelberg, Germany; ^3^ Biochemistry Center (BZH), Heidelberg University, Heidelberg, Germany; ^4^ Section for Cell Biology and Physiology, Department of Biology, University of Copenhagen, Copenhagen, Denmark; ^5^ Institute for Ophthalmic Research, Eberhard Karl University of Tübingen, Tübingen, Germany; ^6^ School of Biological Sciences, NTU Institute of Structural Biology, Nanyang Technological University, Singapore City, Singapore

**Keywords:** cilia, cilia ubiquitination, ciliopathies, caveolae, ESCRT, clathrin-mediated endocytosis, ciliary proteostasis

## Abstract

Establishment and maintenance of the primary cilium as a signaling-competent organelle requires a high degree of fine tuning, which is at least in part achieved by a variety of post-translational modifications. One such modification is ubiquitination. The small and highly conserved ubiquitin protein possesses a unique versatility in regulating protein function *via* its ability to build mono and polyubiquitin chains onto target proteins. We aimed to take an unbiased approach to generate a comprehensive blueprint of the ciliary ubiquitinome by deploying a multi-proteomics approach using both ciliary-targeted ubiquitin affinity proteomics, as well as ubiquitin-binding domain-based proximity labelling in two different mammalian cell lines. This resulted in the identification of several key proteins involved in signaling, cytoskeletal remodeling and membrane and protein trafficking. Interestingly, using two different approaches in IMCD3 and RPE1 cells, respectively, we uncovered several novel mechanisms that regulate cilia function. In our IMCD3 proximity labeling cell line model, we found a highly enriched group of ESCRT-dependent clathrin-mediated endocytosis-related proteins, suggesting an important and novel role for this pathway in the regulation of ciliary homeostasis and function. In contrast, in RPE1 cells we found that several structural components of caveolae (CAV1, CAVIN1, and EHD2) were highly enriched in our cilia affinity proteomics screen. Consistently, the presence of caveolae at the ciliary pocket and ubiquitination of CAV1 specifically, were found likely to play a role in the regulation of ciliary length in these cells. Cilia length measurements demonstrated increased ciliary length in RPE1 cells stably expressing a ubiquitination impaired CAV1 mutant protein. Furthermore, live cell imaging in the same cells revealed decreased CAV1 protein turnover at the cilium as the possible cause for this phenotype. In conclusion, we have generated a comprehensive list of cilia-specific proteins that are subject to regulation *via* ubiquitination which can serve to further our understanding of cilia biology in health and disease.

## Introduction

Protruding from the surface of almost all vertebrate cell types, primary cilia are dynamic microtubule-based cellular antennae with a wide range of signaling functions, varying from mechano-sensation to photoreception (Anvarian et al., 2019). Despite its small volume (Mukhopadhyay et al., 2017), the primary cilium is a uniquely organized microcompartment ideally suited to detect extracellular cues and relay these in order to induce downstream changes in the cell body. Its microtubule-based cytoskeleton, the ciliary axoneme, extends from a modified mother centriole called basal body and projects into the extracellular space. It serves both as a length determinant, as well as a track for the bi-directional movement of intraflagellar transport (IFT) protein complexes (IFT-A and IFT-B) and its retrograde membrane protein cargo adaptor module, the BBSome (Nachury and Mick 2019; Pigino 2021). Although continuous with the plasma membrane, the membrane surrounding the ciliary axoneme has a distinct composition of proteins and lipids owing to the presence of the transition zone at the ciliary base, which functions as a gating structure regulating ciliary protein entrance and exit (Garcia-Gonzalo and Reiter 2017; Garcia et al., 2018). Furthermore, the periciliary membrane compartment that lies at the interface between the ciliary and plasma membranes may also contribute to ciliary membrane compartmentalization. In some cell types, such as fibroblasts and RPE1 cells, the periciliary membrane is invaginated to form the ciliary pocket (CiPo) surrounding the proximal region of the cilium (Benmerah 2013).

Cilia are subject not only to spatial but also to strict temporal regulation. For example, in cycling cultured mammalian cells primary cilia are only present during the G1/G0 phases. Upon cell cycle re-entry, cilia are disassembled to release the centrosomal centrioles for mitotic spindle pole formation (Wang and Dynlacht 2018). Mutations in genes that lead to defects in structure or function of the cilium give rise to a broad and heterogeneous spectrum of overlapping genetic disorders called ciliopathies. Phenotypes caused by ciliary dysfunction include polycystic kidney disease, retinal degeneration, hearing loss, obesity, skeletal and brain abnormalities (Reiter and Leroux 2017).

The structural dynamics and signaling function of primary cilia are regulated by a variety of posttranslational modifications (PTMs) such as lipidation (Roy and Marin 2019), phosphorylation and ubiquitination (May et al., 2021b). Ubiquitin is a highly conserved protein of only 8.5 kDa size which possesses the uniquely versatile property of being able to form monomeric, as well as polymeric chains of different topologies onto target proteins. This is achieved through the seven lysine (K) residues in the ubiquitin protein (K6, K11, K27, K29, K33, K48, K63) that can each serve as a basis for the formation of polyubiquitin chains of either uniform or mixed nature. A different outcome awaits target proteins depending on the type of polyubiquitin chain attached. The most abundant modification, K48-linked chains, marks proteins for degradation by the proteasome, while K63 polyubiquitin chains are associated with both proteolytic and non-proteolytic functions (Komander and Rape 2012). Ubiquitination is a three-step enzymatic cascade, which begins with the ATP-dependent activation of ubiquitin by an E1-activating enzyme and its subsequent transfer onto an E2-conjugating enzyme. Finally, ubiquitin is attached to proteins *via* a substrate specific E3 ligase enzyme. Ubiquitination is a reversible modification. Removal of ubiquitin is achieved by deubiquitinating (DUB) enzymes which are also substrate specific. The obvious complexity of the “ubiquitin code” delivered by means of chains of varying length and topology, combined with specific “readers” and “erasers” of this code allow for a high degree of fine-tuning (Komander and Rape 2012).

Immunogold labeling revealed the presence of ubiquitin in the cilia of ductuli efferentes as early as 1996 (Fraile et al., 1996), but the importance of cilia-specific ubiquitination took over a decade longer to gain the spotlight. Since then, ubiquitination has been implicated in regulation of cilium assembly and disassembly, regulation of signaling pathways conducted through the cilium, and regulation of ciliary protein content.

In 2009, Huang et al. reported that levels of ubiquitinated proteins increase upon ciliary resorption in *Chlamydomonas*, and this accumulation is even more pronounced in an IFT mutant background, suggesting that the IFT machinery is important in trafficking of ubiquitinated cargo (Huang et al., 2009). Specifically, out of the 20 proteins which become ubiquitinated during the disassembly of cilia, α-tubulin modified at K304 was the most abundant and this modification was required for ciliary resorption (Wang et al., 2019b). More recently, it has been shown that mutations in the IFT-associated BBSome complex members result in the aberrant accumulation of proteins in several types of cilia (Wingfield et al., 2018). For example, defects in BBS genes lead to accumulation of several GPCRs in cilia of cultured IMCD3 cells, as these GPCRs are normally ubiquitinated for BBS-mediated retrieval from cilia (Shinde et al., 2020; Shinde et al., 2022). Similarly, the outer segments of BBS mutant mouse retina accumulate ubiquitinated substrates, causing retinal degeneration (Datta et al., 2015; Shinde et al., 2020; Shinde et al., 2022).

Inactivation of Aurora-A mediated by the removal of Trichoplein from the mother centrioles is required at the initial stages of ciliogenesis (Goto et al., 2013). Trichoplein becomes polyubiquitinated by KCTD17, a substrate adaptor for the Cul3-RING E3 ligases, which allows axonemal extension (Kasahara et al., 2014). Several lines of research have indicated a critical role for the K63-deubiquitiunase CYLD in regulating ciliogenesis. CEP70 and CEP350 both rely on the DUB activity of CYLD for their proper localization to the centrosome. Inhibition of cilia formation upon exogenous overexpression of CYLD corroborates its function as a negative regulator of ciliogenesis (Eguether et al., 2014; Yang et al., 2014). Conversely, CEP350 recruits CEP78 to the mother centriole, leading to activation of the E3 ligase UBR5 that ubiquitylates CP110, a key negative regulator of ciliogenesis, thereby promoting CP110 removal to initiate ciliogenesis (Goncalves et al., 2021; Hossain et al., 2017). The quintessential centriolar satellite protein, PCM1, is heavily regulated by the ubiquitin proteasome system. PCM1 is mono-ubiquitinated by the E3 ligase MIB1, which contributes to maintaining centriolar satellite structures under non-stressed conditions and suppressing ciliogenesis (Wang et al., 2019b; Villumsen et al., 2013). MIB1 is tethered to the centriolar satellites by binding to PCM1, which it can also polyubiquitinate and thus mark for proteasomal degradation, resulting in destabilization of the centriolar satellites. This tethering also prevents recruitment of MIB1 to the ciliary base to poly-ubiquitinate TALPID3 and suppress ciliogenesis (Wang et al., 2016). CYLD has been shown to regulate the levels of MIB1 and thus contribute to inhibition of PCM1 degradation (Douanne et al., 2019). Another DUB, USP9X, similarly antagonizes MIB1-mediated loss of PCM1 (Han et al., 2019; Wang et al., 2019a). Interestingly, USP9X mutations are causative of a female specific syndrome which manifests with many hallmark ciliopathy phenotypes such as skeletal defects, polydactyly, brain abnormalities and developmental delay (Reijnders et al., 2016; Homan et al., 2014). As USP9X is also a DUB for the ciliary and ciliopathy-associated protein NPHP5/IQCB1 protein, resulting in its stabilization and maintenance of cilium function, disruption of USP9X by genetic mutation could induce the opposite effect (Das et al., 2017).

The ciliary membrane is enriched for sensory receptors whose adequate processing is crucial to faithful regulation of downstream signaling cascades. Upon ligand binding, the Patched 1 (PTCH1) receptor exits cilia and the GPCR Smoothened (SMO) accumulates inside cilia to signal activation of the Hedgehog signaling pathway, a well-described ciliary-mediated signaling pathway (Anvarian et al., 2019; Wang et al., 2016). Desai et al. demonstrated that in the absence of ligand binding, SMO is continuously ubiquitinated and removed from cilia in an IFT27/BBS-dependent manner (Desai et al., 2020). Furthermore, the BBSome-dependent removal of GPR161 from the cilium, a negative regulator of Hedgehog signaling, is also regulated *via* K63-dependent ubiquitination. In a similar fashion, activation of somatostatin signaling is followed by ciliary exit of the ubiquitinated SSTR3 receptor (Shinde et al., 2020). Canonical Wnt signaling acts through strict regulation of β-catenin levels. The E2 conjugating enzyme UBE2E1, which mediates polyubiquitination of β-catenin was shown to bind the ciliopathy-associated protein MKS1. Loss of UBE2E1 recapitulated ciliary defects likely caused by Wnt-signaling defects (Szymanska et al., 2022).

Collectively these reports highlight the significant contribution of ubiquitination in ciliary assembly, maintenance and function. The aim of our study was to develop unbiased proteomics approaches focusing on generating a blueprint of the ciliary ubiquitinome: a comprehensive list of cilia-specific protein modules and processes which are regulated by ubiquitination.

To this end, we used ciliary targeted ubiquitin to perform affinity proteomics in RPE1 cells and ubiquitin-binding domain proximity labeling proteomics in IMCD3 cells. Our data suggest an important contribution of the ESCRT-dependent clathrin-mediated endocytic pathway in regulating ubiquitination dependent processes in the latter. In RPE1 cells, caveolae and specifically ubiquitination of CAV1 were found to be implicated in regulating ciliary length.

## Materials and methods

### Cloning

Human NPHP3 (aa 1-203) (ENSG00000113971) was used as a ciliary targeting signal in both IMCD3 and RPE1 cells, and hereafter referred to as NPHP3. NPHP3-HA-Ubiquitin (ENST00000339647.6), or NPHP3-HA-Ubiquitin K0 (G75A-G76A, all Lys mutated to Ala, and C-terminal Gly mutated to Ala) fusions were incorporated into a Gateway pDONR201 vector. Using traditional Gateway cloning (Thermo Fisher Scientific) the ubiquitin sequences (kindly donated by Dr. Bert van der Reijden) were cloned into pDONR201 *via* a BP reaction (following the manufacturer’s instructions), while the NPHP3 and HA sequences were inserted using a PCR-based large insert cloning strategy. The NPHP3-HA-Ubiquitin fusions were further subcloned into a Gateway destination vector *via* an LR reaction.

The C terminal UBA domains (aa 185–409) of human RAD23B (ENST00000358015.8) (kindly provided by Dr. David P Toczyski (Mark et al., 2016), were cloned into pDONR201 serving as a polyubiquitin ligase trap. The BioID2 sequence was obtained from Addgene (MCS-BioID2-HA was a gift from Kyle Roux; Addgene plasmid #74224; http://n2t.net/addgene:74224; RRID:Addgene_74224) (Kim et al., 2016) and incorporated into a pgLAP1 vector *via* PCR-based large insert cloning. Subsequently, NPHP3 was inserted upstream of BioID2 using the same strategy to create a pgLAP1-NPHP3-BioID2 Gateway destination vector suitable for N-terminal tagging. This plasmid was used to perform an LR reaction with pDONR201-RAD23B (aa 185–409).

CAV1 wild type (ENST00000341049.7) and CAV1 lysine-less mutant sequences, previously described (Kirchner et al., 2013) and kindly provided by Dr. Hemmo Meyer, were cloned into pDONR201 and subsequently subcloned into a pgLAP5 vector (pgLAP5 was a gift from Peter Jackson; Addgene plasmid # 19706; http://n2t.net/addgene:19706; RRID:Addgene_19706) to create C-terminal eGFP fusions.

RAB8A and ARL13B with N-terminal mCherry fusion were generated as Gateway Entry plasmids (pENT220) using standard molecular biology techniques. Subsequently, lentiviral transfer vectors were generated by recombination into Gateway Destination vector pCDH.EF1A.GW.IRES.Blast (gift of Kay Schink) (Campeau et al., 2009) *via* LR reaction. Lentiviral particles were generated in HEK293Tcells using second generation lentiviral packaging vectors pMD2.G and pCMVΔ-R8.2 (kindly provided by Carlo Rivolta).

### Cell line generation

Murine inner medullary collecting duct 3 (IMCD3) Flp-In cells (a kind gift from Dr. Maxence Nachury) were stably transfected using the Thermo Fisher Scientific Flp-In™ technology to express NPHP3-BioID2 (hereafter called BioID2-control) or NPHP3-BioID2-RAD23B (aa185-409) (hereafter called BioID2-Uiquitin Binding Domain, BioID2-UBD). In summary, cells were transfected with equal amounts of plasmid DNA encoding the gene of interest and the Flp-In recombinase pOG44 using Lipofectamine 2000 (Life technologies; 11668019), followed by selection with 400 ug/ml Hygromycin (Sigma-Aldrich; H3274).

Human TERT-immortalized retinal pigment epithelial 1 (RPE1) cells were stably transfected with plasmids expressing NPHP3-HA-Ubiquitin or NPHP3-HA-Ubiquitin K0. Briefly, cells were transiently transfected with linearized plasmid DNA using electroporation (Amaxa Cell Line Nucleofactor Kit V, cat #VCA-1003), selected with G418 geneticin (Sigma-Aldrich; G8168), and single cell sorted to generate monoclonal lines.

RPE1 Flp-In cells were obtained from Ximbio (cat # 153242) and stably transfected with plasmids coding for wiltype CAV1-eGFP or mutant CAV1-eGFP as described. Stable transfectants were selected using 500 ug/ml Hygromycin.

### Immunofluorescence microscopy analysis

RPE1, RPE1 Flp-In, and IMCD3 Flp-In cells were cultured in DMEM (Dulbecco’s Modified Eagle’s Medium, Sigma-Aldrich, D0819): F12 (Ham′s Nutrient Mixture F12, Sigma-Aldrich, N6658) 1:1, supplemented with 10% Fetal Calf Serum (Sigma-Aldrich, F0392) or 0.2% in the case of starvation medium, 1% Sodium Pyruvate (Sigma-Aldrich, S8636) and 1% Penicillin-Streptomycin (Sigma-Aldrich, P4333). To induce cilia formation, cells were grown in starvation medium for 48 hrs. Shortly, cells were washed in PBS, then fixed and permeabilized in either 2% paraformaldehyde (PFA) for 20 min, or ice-cold methanol for 5 min, followed by extensive washing with PBS. After blocking in 5% Bovine Serum Albumin (Sigma-Aldrich, A6003), cells were incubated with primary antibodies for 1.5 hrs at room temperature. The following primary antibodies were used: mouse anti-HA (1:1000, Sigma-Aldrich clone HA-7, H9658), chicken anti-BioID2 (1:1500, BioFront Technologies, BID2-CP-100), or Streptavidin-Alexa 488 conjugate (1:1000, Thermo Fisher Scientific, S32354), rabbit anti-CAV1 (1:500, Proteintech, 16447-1-AP), and rabbit anti-CAVIN1 (1:500 Cell Signaling Technology, clone D8C1D, 46379). In addition, rabbit anti-ARL13B antibodies (1:500, Proteintech, 17711-1-AP) were used to mark cilia, guinea pig anti-RPGRIP1L (1:500, home-made) was used as a ciliary transition zone marker, mouse anti-PCM1 (1:500, Santa Cruz, SC-398365) was used to stain centriolar satellites, and rabbit anti-EHD1 (1:500, Abcam, ab109311) was used to label the ciliary pocket. To wash off the primary antibodies, cells were extensively washed in PBS with 0.05% Tween (PBST). Subsequently, cells were incubated with secondary antibodies, Alexa Flour 488 (1:800, Invitrogen), Alexa Fluor 568 (1:800, Invitrogen), and Alexa Fluor 647 (1:800, Invitrogen) for 45 min followed by washing with PBST. Finally, cells were shortly rinsed in deionized water and samples were mounted using Vectashield with DAPI (Vector Laboratories, H-1200). Images were taken using an Axio Imager Z2 microscope with an ApoTome (Zeiss) at 63x magnification.

### Live cell imaging

For live cell imaging, cells were seeded on glass-bottom dishes (35/22 mm; HBSt-3522; WillCo Wells) and serum-starved the next day. Imaging was performed after 24–48 hrs of serum-starvation. Before imaging, medium was replaced by phenol-free medium DMEM. For SiR-Tubulin experiments, cells were incubated with phenol-free DMEM containing 1 µM SiR-Tubulin-Cy5 probe for 1 h. Imaging was performed under regulated temperature (37°C) and humidity (5%) on a fully motorized Olympus IX83 inverted microscope equipped with a spinning disk (Yokogawa) and a Hamamatsu ORCA-Flash 4.0 digital camera (C13440) with 100× numerical aperture (NA) 1.4 oil objective (Olympus). The 488 nm, 561 nm and 640 nm laser lines were used for imaging eGFP, mCherry and SiR-Tubulin-Cy5, respectively. The time-lapse sequences ranged from 5 to 10 s.

### Transmission electron microscopy

RPE1 cells were processed for conventional TEM as follows: cells were grown on 35 mm glass bottom dishes (MatTec Cooperation), serum-starved for 48 h, and fixed with 2.5% glutaraldehyde (EM grade, EMS) in 0.1 M cacodylate buffer (CB, EMS) pH 7.4 for 1 h on ice. Cells were post-fixed in 1% osmium tetroxide (EMS) in CB, incubated with 1% low molecular weight tannic acid (EMS) in CB for 30 min at room temperature, and stained *en bloc* with 2% uranyl acetate in distilled water overnight. Specimen were dehydrated using a graded ethanol series and embedded in Durcupan resin (Sigma-Aldrich). Cells were sectioned parallel to the substratum using a diamond knife. 70–80 nm semithin sections were picked up on formvar- and carbon-coated EM slot grids and imaged on a TecnaiT12 TEM (Thermo Fisher Scientific) operated at 120 kV equipped with a 4k × 4k Eagle camera (Thermo Fisher Scientific). APEX labeling on the CAVIN1-APEX2-eGFP RPE1 cell line was performed as described previously (Ludwig 2020).

### Western blot analysis

SDS-PAGE and western blotting were performed using the NuPAGE system from Thermo Fisher Scientific according to the manufacturer’s instructions. Proteins expressing the HA-tag were detected using a mouse monoclonal anti-HA antibody (1:1000, Sigma-Aldrich, clone HA-7). Recombinant proteins carrying a BioID2-tag were identified *via* chicken polyclonal anti-BioID2 antibodies (1:5000, BioFront Technologies, BID2-CP-100). For immunodetection of proximity labelled proteins a Streptavidin-IRD800 conjugate (1: 10000, Li-cor, 926–32230) was used and the blots were analyzed on an Odyssey DLx (Li-cor).

### BioID2 proximity labelling

Proximity labelling was performed using the BioID2-UBD line described above, with a BioID2-control as negative control. Cells were plated at a confluency of 15% and cultured for 24 h in normal medium containing DMEM/F12 1:1 with 10% FCS and supplemented as described above. Subsequently, cells were stimulated for ciliogenesis for 48 h using starvation medium (0.2% FCS). Proximity labelling was induced for the last 24 h by supplementing the medium with 10 μM Biotin (Sigma-Aldrich, B4501). The cells were lysed in RIPA buffer (50 mM Tris-HCl pH 7.5, 150 mM NaCl, 1% NP-40, 0.1% SDS (Life Technologies, 15553-027), 0.5% Sodium Deoxycholate, 1 mM UltraPure EDTA pH 8.0 (Invitrogen, 11568896), followed by sonication. Cell lysis was continued for 1 h at 4°C while rotating. Lysates were cleared by centrifugation at 14,000 rpm for 20 min at 4°C, snap frozen and at −80°C stored until sample enrichment. Sample enrichment for biotinylated substrates was performed for 3 h at 4°C using StrepTactin Superflow beads (IBA, 2-1206-025). After incubation the beads were washed two times in 4°C 1xTBS (20 mM Tris, 150 mM NaCl) prior to on-bead digestion for 1 h at 27°C and 800 rpm, in Trypsin digestion buffer (2 mM Urea, 50 mM Tris-HCl (pH 7.5), 5 μg/sample trypsin (Serva, 9002-07-7). After digestion the beads were washed two times in digestion washing buffer (2 mM urea, 50 mM Tris-HCl (pH 7.5), 1 mM DTT). The on-bead digestion solution and two bead washes were pulled per sample to be snap frozen and stored at −80°C until mass spectrometry (MS) analysis.

### HA-pulldown

RPE1 cells stably expressing the ubiquitin variants described above were seeded at 40% confluency and cultured in normal medium for 24 h, prior to ciliogenesis induction with starvation medium for an additional 48 h. The HA-based ubiquitin pulldown was performed under non-denaturing conditions. Cell lysis was performed as described above for the BioID2 samples, snap frozen and stored at −80°C until HA-purification. After thawing on ice, HA-purification was performed for 3 h at 4°C using anti-HA affinity beads (Thermo Fisher Scientific, 26181). After incubation the beads were washed, and an on-bead digestion was performed as described above for the BioID2 samples. Sample were snap frozen and stored at −80°C until MS analysis.

### Mass spectrometry analysis and statistical identification of enriched proteins

The trypsinized HA pull down and BioID2 proximity labelling samples were analysed on the Q Exactive Plus mass spectrometer as described in (Beyer et al., 2021). Next, Label-free quantification (LFQ) was performed, using Maxquant (V.1.6.1.0) (Cox and Mann 2008; Cox et al., 2014; Beyer et al., 2021). In this, protein identification was done using a human or mouse Swissprot database subsets form November 2019 (20,367 protein entries) or August 2019 (17,027 protein entries) respectively. Identified proteins were further analyzed for statistical enrichment. Identified proteins were classified in a three-Tier system. Tier 3 proteins were filtered to be present in at least 66% of the replicates for the sample of interest (excluding control samples). From these, Tier 2 proteins were classified if they were also Significance A (SignA) positive as calculated in R (version 4.0.4) (R Core Team 2021). Significant proteins were identified using a one-sided SignA outlier test with a Benjamini–Hochberg FDR correction, performed as described in Cox and Mann, SignA ≤0.05 (Cox and Mann 2011). Of these Tier 2 proteins, Tier 1 proteins were identified as being also Welch’s *t*-test positive. For this, LFQ ratios were used in a one-sided paired Welch’s *t*-test in combination with a Benjamini–Hochberg *p*-value correction, both from the stats package in R, with a q-value ≤0.05.

### Pathway enrichment analysis and active subnetwork identification

Tier 1 significant proteins per analyzed dataset were used for pathway enrichment analysis. When required, mouse gene IDs were translated to human gene IDs to allow comparison of the different dataset. Translation was performed using the homologene package from R (Mancarci 2019). GO Term enrichment was performed in three sets: biological processes (BP), molecular functions (MF), and cellular component (CC). Enriched terms were identified using the TopGo package in R, nodesize = 3, filtered for an Fisher’s exact test value ≤0.05 (Alexa and Rahnenfuhrer 2020).

The combined data from the UBD-PL and UAP approaches was used for active subnetwork identification using DOMINO, as previously described (Levi et al., 2021). Active subnetworks were scored based on STRING interaction data. Networks were displayed using Cytoscape (v.3.9.1.) (Shannon et al., 2003).

### Ciliation and ciliary length analysis

To determine percentage of ciliated cells and ciliary length, three biological replicates and three technical replicates were performed. For both analyses, ARL13B was used a cilia marker and ALPACA was used for automated image analysis of the cilium phenotype (Doornbos et al., 2021). Statistical significance was calculated using One-way ANOVA with a Kruskal–Wallis test (GraphPad Prism software). Four asterisks indicate significance *p*-value < 0.0001. Error bars indicate mean with SD.

## Results

### Establishing and validating model systems to study cilia-specific ubiquitination

To study cilia-specific ubiquitination, two different proteomics approaches were developed based on either affinity purification or proximity labeling. In both instances, we used a previously described ciliary targeting method utilizing amino acids 1-203 of the NPHP3 protein as an efficient ciliary localization signal (Mick et al., 2015).

**FIGURE 1:**
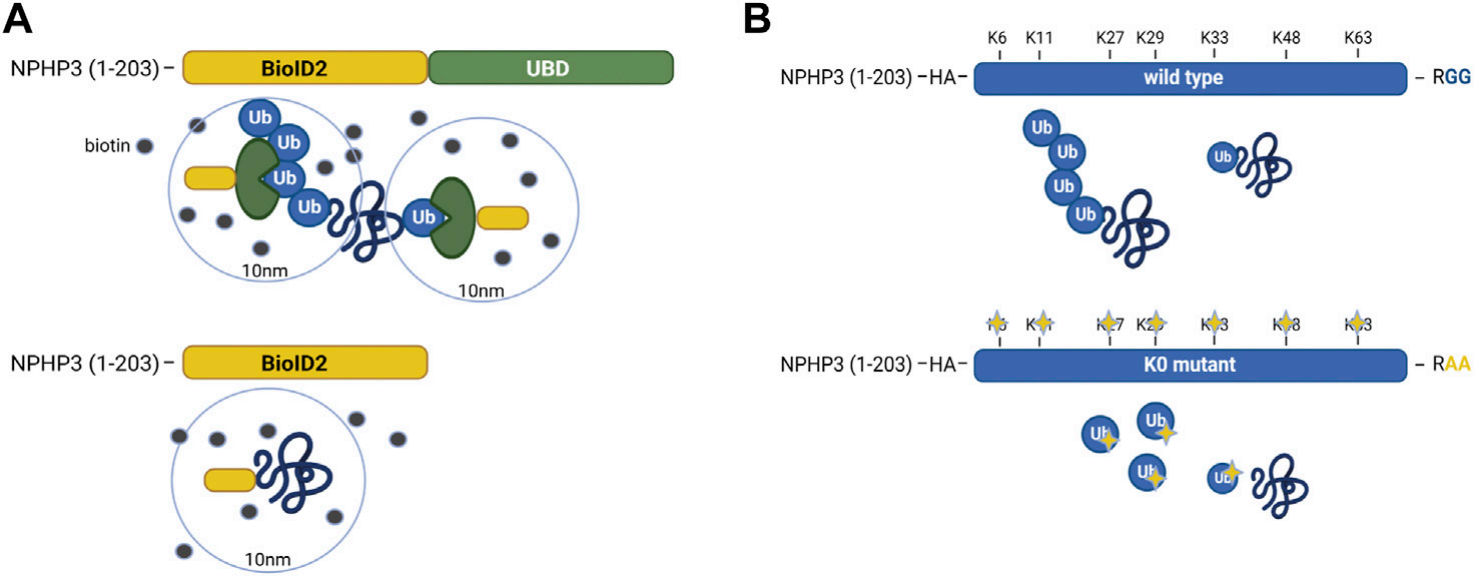
Schematic representation of the UBD-PL and UAP proteomics approaches **(A)** In IMCD3 cells, the Ubiquitin-binding domain Proximity Labeling (UBD-PL) approach was developed. Using the NPHP3 targeting strategy, the C-terminal ubiquitin-binding domain (UBD) of the RAD23b protein was fused to the BioID2 proximity labeling tag. The RAD23b protein contains a tandem of two UBAs at its C terminus, which were utilized in this study as an indirect bait for polyubiquitinated substrates. These polyubiquitinated targets will become biotinylated when they come into proximity with the BioID2 tag, which promiscuously attaches biotin moieties to primary amines within an estimated radius of 10 nm. As a control, an NPHP3 (1-203)-targeted BioID2 recombinant protein was used. **(B)** For the Ubiquitin Affinity Proteomics (UAP) approach in RPE1 cells, the NPHP3 ciliary targeting sequence was fused to either wild type (WT) ubiquitin, or a lysine-less ubiquitination-impaired K0 ubiquitin mutant which is unable to bind substates, as the C-terminal Gly residues were mutated to Ala. To facilitate affinity purification and visualization in cells, the hemagglutinin (HA) tag was included at the N-terminus of ubiquitin in both recombinant proteins. The ubiquitination-impaired K0 will not be able to bind nor substrates, nor ubiquitin.

**FIGURE 2:**
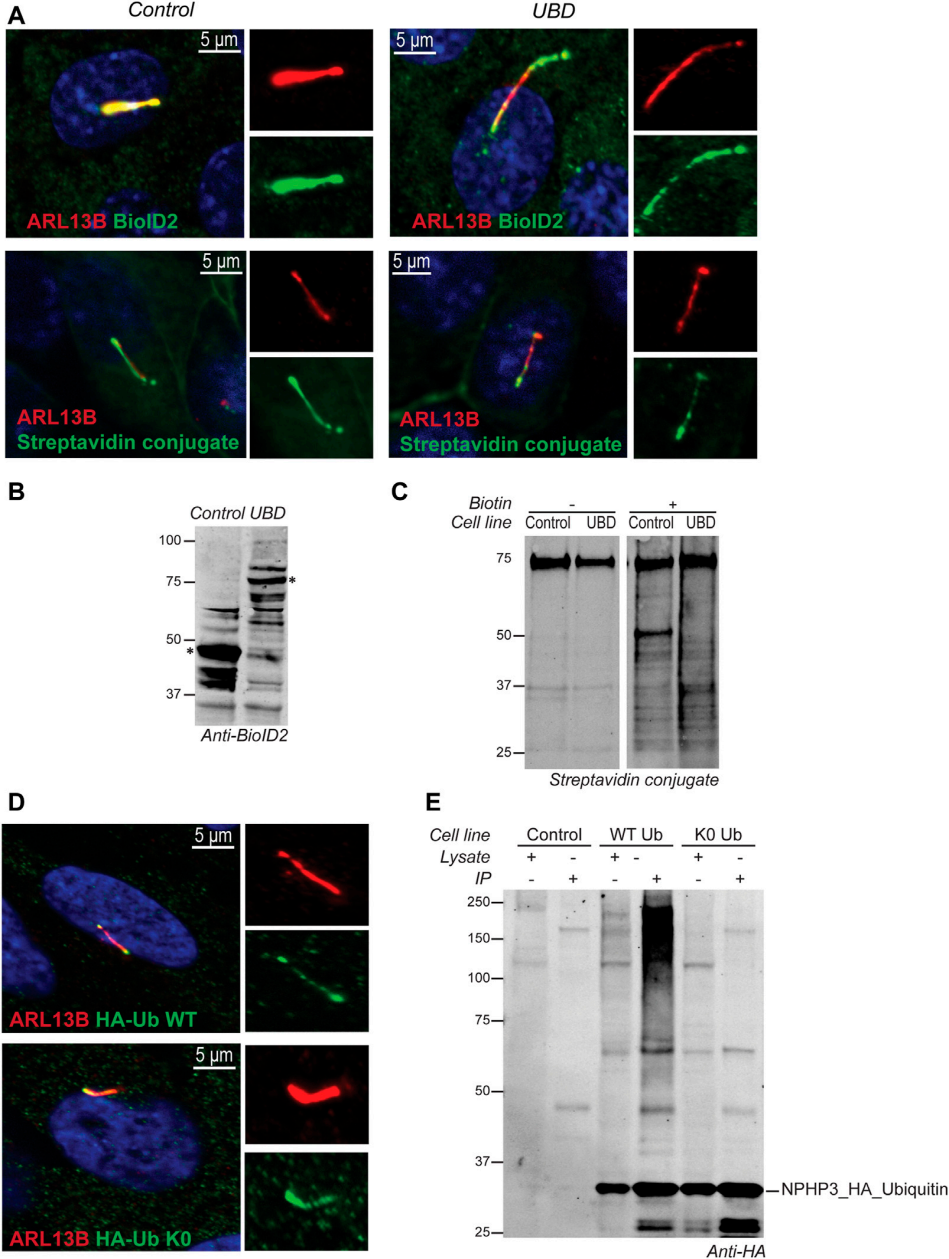
Validation of model systems to study the ciliary ubiquitinome. **(A)** IF localization of the UBD and empty vector control BioID2 constructs in stable IMCD3 Flp-In cells. Both proteins colocalized with ARL13B (red) at the primary cilium when co-stained with an antibody against the BioID2 tag (green; upper panels). Upon initiation of proximity labeling, biotinylated substrates can be detected in the cilium (red) using a conjugated Streptavidin antibody (green; lower panels). **(B)** A western blot stained with anti-BioID2 was used to verify correct expression of both constructs in whole cell extracts of IMCD3 Flp-In cells. **(C)** Immunoprecipitation upon addition of biotin showed a ladder of biotinylated proximal targets in both control and UBD cells when labeled with a conjugated Streptavidin antibody. **(D)** IF analysis of stably transfected RPE1 cells confirmed that both the wild type ubiquitin (HA-Ub WT), as well as the ubiquitination impaired mutant (HA-Ub K0) detected using an anti-HA staining (green) colocalized with ARL13B (red) at the primary cilium. **(E)** Immunoblotting using an anti-HA antibody indicated that both ubiquitin variants were expressed correctly compared to untransfected control cells. The recombinant proteins and their respective interactors could successfully be purified from lysates by immunoprecipitation. Furthermore, as expected, the immunoprecipitated HA- Ub WT also showed a characteristic smear of ubiquitinated substrates at high molecular weight which was not observed for the ubiquitination impaired HA- Ub K0 mutant demonstrating that polyubiquitin chain formation is not impeded in the HA-Ub WT cells.

In IMCD3 cells, the Ubiquitin-binding domain Proximity Labeling (UBD-PL) approach was developed (Figure 1A). Using the NPHP3 targeting strategy, the C-terminal ubiquitin-binding domain (UBD) of the RAD23B protein was fused to the BioID2 proximity labeling tag (Kim et al., 2016). UBDs are small modular domains which can form non-covalent bonds with ubiquitin (Dikic et al., 2009). Ubiquitin-associated domains (UBAs) are among the most common types of UBDs. The RAD23B protein contains a tandem of two UBAs at its C terminus (Chen et al., 2001), which was utilized in this study as an indirect bait for polyubiquitinated substrates. These polyubiquitinated targets will become biotinylated when they come in close proximity with the BioID2 tag, which promiscuously attaches biotin moieties to primary amines within an estimated radius of 10 nm (Kim et al., 2016). As a control, an NPHP3 (1-203)-targeted BioID2 recombinant protein was used. Immunostaining of the control and bait fusion proteins revealed accurate localization of both proteins to the ciliary compartment, as confirmed through co-localization with ARL13B (Figure 2A, upper panels). The same was true for the proximally biotinylated targets upon overnight treatment with biotin (Figure 2A, lower panels). Both recombinant proteins were detected at the expected size on a western blot (Figure 2B). Furthermore, biotin supplementation, followed by immunoprecipitation and western blotting, indicated a ladder of biotinylated substrates of various sizes confirming the presence of proximity labeled proteins in the samples (Figure 2C).

For the Ubiquitin Affinity Proteomics (UAP) approach in RPE1 cells, the NPHP3 ciliary targeting sequence was fused to either wild type (WT) ubiquitin, or a lysine-less ubiquitination-impaired K0 ubiquitin mutant which is unable to become conjugated to substrates and build polyubiquitin chains, as the C-terminal Gly residues were mutated to Ala (Figure 1B). To facilitate affinity purification and visualization in cells, the hemagglutinin (HA) tag was included at the N-terminus of ubiquitin in both recombinant proteins. Both ubiquitin variants were correctly localized to the cilium when transfected cells were co-stained with an antibody against the ciliary membrane marker ARL13B (Figure 2D). Moreover, immunoprecipitated WT ubiquitin showed the characteristic high molecular weight “smear” indicative of polyubiquitination of target substrates, whereas the ubiquitin K0 mutant did not (Figure 2E).

Altogether, we conclude that both the UBD-PL, as well as the UAP-based proteomics approaches, should yield cilia-specific polyubiquitinated proteins in transfected IMCD3 and RPE1 cell lines, respectively.

### Cilia-specific ubiquitinome of IMCD3 cells suggests a role for ESCRT-dependent clathrin-mediated endocytosis in the regulation of ciliary function

Mass spectrometry analysis following biotinylation of the cilia targeted UBDs in IMCD3 Flp-In cells resulted in the identification of a total of 1650 proteins across 6 experimental replicates per cell line. These were further subdivided into three Tiers based on stringency criteria. Tier 3 contained all the proteins which were not significantly enriched. Tier 1 (q-value ≤0.05 & Sign. A ≤ 0.05) comprised 70 highly significantly enriched proteins, whereas an additional 59 proteins were present in Tier 2 (*p*-value ≤0.05) when a less stringent cut-off was applied (Supplementary Table S1). RAD23B, which was used as a bait in this context, was found to be the most highly enriched protein in the dataset, thus technically validating the approach.

**FIGURE 3:**
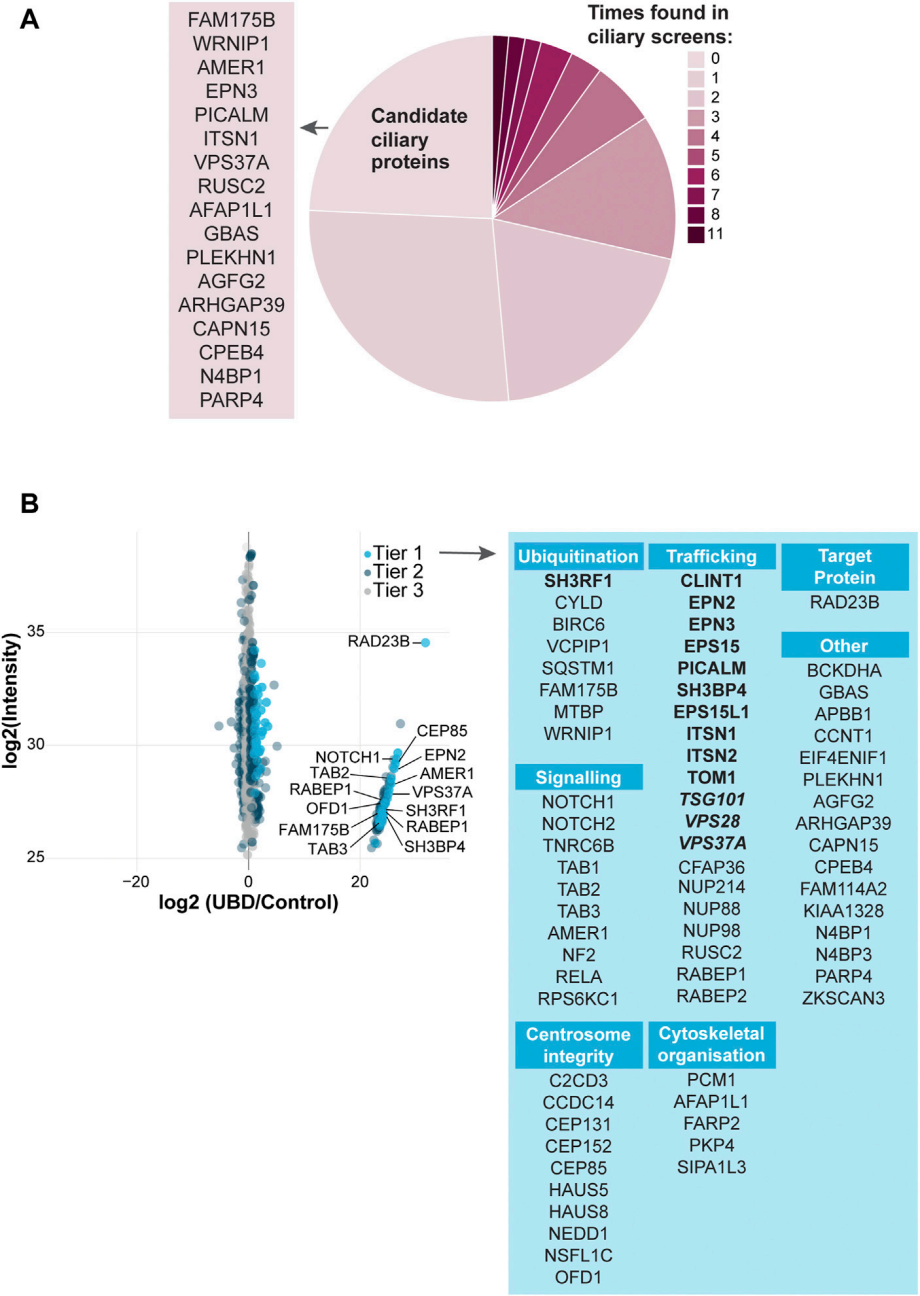
Analysis of UBD mass spectrometry results **(A)** Comparison of the significantly enriched proteins from tier 1 with published ciliary-datasets. Most proteins were previously identified in other ciliary studies, of which OFD1, C2CD3, PCM1, CEP131, and SQSTM1 were identified most often (dark pink). The remaining proteins are indicated as “New candidate ciliary proteins” on the left of the pie plot **(B)** MS analysis of enriched proteins in the UBD *versus* the control cell line after proximity labelling. The proteins are colored according to their significance tier. All Tier 1 significant proteins were grouped by function (light blue), for which the clathrin-related proteins (bold) and ESCRT proteins (bold italic) are highlighted. The complete list of identified proteins (Tier 1, 2, and 3) can be found in Supplementary Table S1.

To test the specificity of our UBD-PL approach, we compared our dataset to a variety of ciliary screens and databases generated over the last decade (Sang et al., 2011; Ishikawa et al., 2012; Nakata et al., 2012; van Dam et al., 2013; Firat-Karalar et al., 2014; Gupta et al., 2015; Mick et al., 2015; Roosing et al., 2015; Wheway et al., 2015; Kim J. H. et al., 2016; Boldt et al., 2016; Toriyama et al., 2016; Kohli et al., 2017; Breslow et al., 2018; Pusapati et al., 2018; van Dam et al., 2019; Lv et al., 2021). We compared the proteins in Tier 1 to these published cilia-specific datasets. 76% of the significantly enriched proteins present in Tier 1 were previously identified in ciliary studies. Among the most frequently found proteins (11 out of 15 included studies) were for instance PCM1, OFD1, C2CD3, CEP131, and SQSTM1. Moreover, CYLD, which is also often present in the aforementioned ciliary screens, is known to regulate PCM1 protein levels *via* its function as a deubiquitinating enzyme (Eguether et al., 2014; Douanne et al., 2019) further validating our approach as specific to both ciliary and ubiquitination-related processes. Several novel candidate ciliary proteins were identified in our study (Figure 3A).

Importantly, we identified a large group of proteins involved in ESCRT-dependent clathrin-mediated endocytosis (Le Roy and Wrana 2005; Shields and Piper 2011; Haglund and Dikic 2012; Kaksonen and Roux 2018; Vietri et al., 2020; Mosesso et al., 2019; Roach et al., 2021; Shinde et al., 2022). The ESCRT-0 STAM2 (Tier 2), and ESCRT-1 TSG101, VPS28, and VPS37A (Tier 1) components, as well as additional members of the clathrin endocytic pathway including SH3RF1, SH3BP4, CLINT1, EPN2, EPN3, EPS15, EPS15L1, PICALM, ITSN1, ITSN2, and TOM1 (Tier1) and TOM1L2, PIK3C2A, AP2B1 (Tier 2) were enriched in our dataset. Clathrin-mediated endocytosis is a major pathway in the internalization and recycling of proteins involved in signaling. Not surprisingly, another significantly enriched group of proteins in our dataset is involved in signaling pathways, such as Wnt, Notch and TGF beta (NOTCH1, NOTCH2, AMER1, TAB1, TAB2, TAB3, TNRC6B, NF2, RELA, and RPS6KC1). A third group of highly enriched proteins in our UBD dataset, consists of proteins involved in centrosomal and centriolar satellite integrity: CEP131, CEP152, CEP85, C2CD3, CCDC14, HAUS5, HAUS8, NEDD1, PCM1, OFD1 (Tier 1) and CEP164, CEP350 (Tier 2) (Figure 3B).

Comprehensive GO term enrichment analysis (Ashburner et al., 2000; Carbon et al., 2021) was performed in order to gain further insight into the specific functional contribution of the proteins identified in our dataset to the regulation of cilia function *via* ubiquitination. This analysis was subdivided into three categories: biological processes (BP), molecular functions (MF), and cellular component (CC).

**FIGURE 4:**
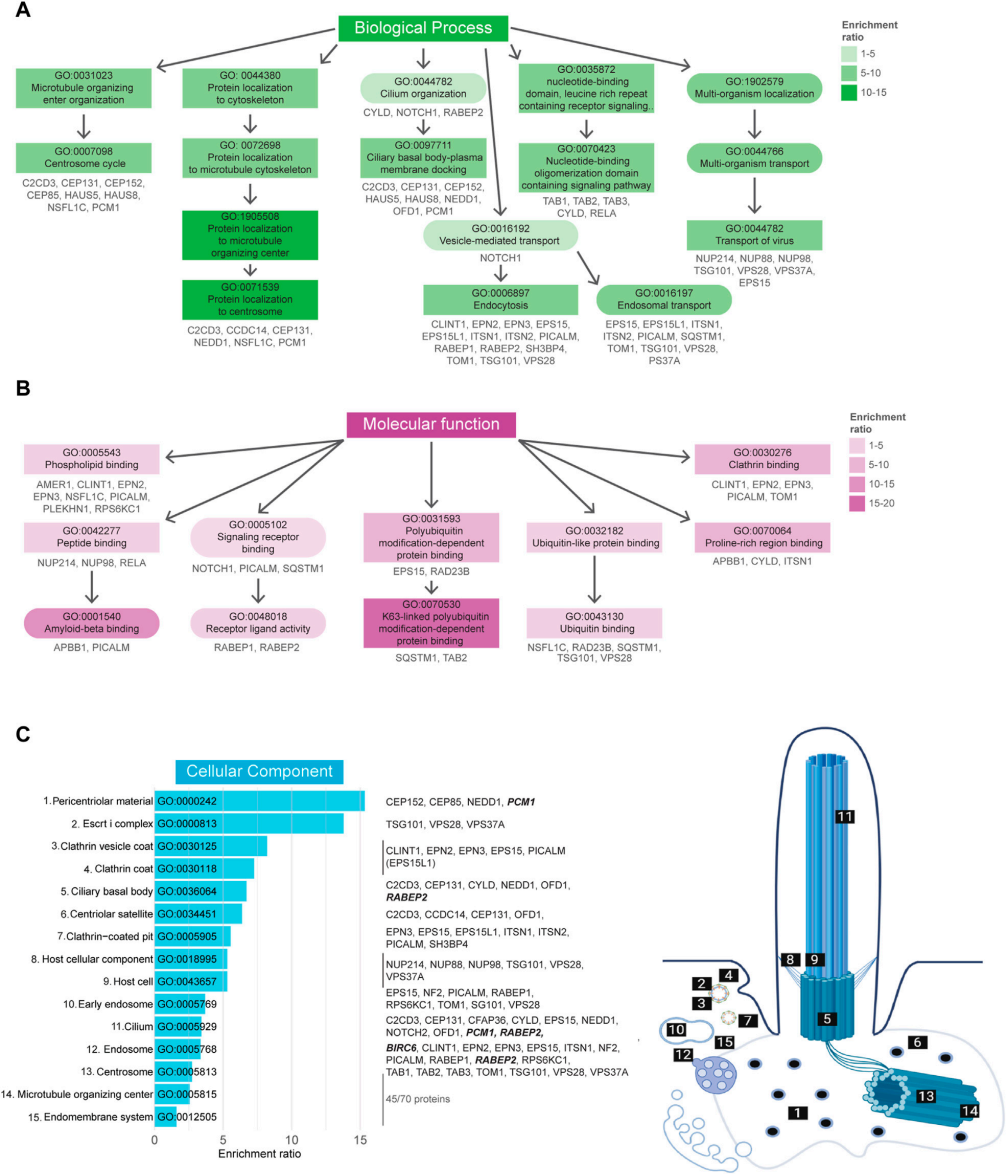
Gene Ontology enrichment analysis of UBD dataset. **(A)** GO term enrichment for biological processes (BP) colored by their enrichment ratio. The 11 most enriched BP terms (Fisher’s exact test value ≤9.5E-5) are displayed (square) along with supporting GO terms (circle). Arrows indicate the related terms and the genes per term are indicated in grey. All upstream terms also include the genes from their downstream terms. **(B)** GO term enrichment analysis for molecular functions (MF) of which the 8 most enriched terms (Fisher’s exact test value ≤5.5E-3) are displayed as in **(B)**. **(C)** The 15 most enriched terms (Fisher’s exact test value ≤7.5E-4) of the cellular components (CC) GO term enrichment analysis are displayed, ordered by their enrichment ratio. The cellular position of these terms and their related proteins are indicated on the right. The terms “Host cellular component” and “Host cell” do not directly refer to a ciliary localization, while their related proteins do (dashed line). The proteins related to the terms ‘Microtubule organizing center’ and ‘Endomembrane system’ were not indicated, as there were too many to clearly display, however a complete list of proteins can be found in Supplementary Table S1. Proteins indicated in bold italic letters were shared hits between UBD-PL and UAP.

A total of 259 BP-related GO terms were significantly enriched in the Tier 1 UBD-based proximity labelling dataset, of which the top 50 most enriched terms can be found in Supplementary Figure S1. Of these, the 11 most enriched BP terms (Fisher’s exact test value ≤9.5E-5) indicate a role for ubiquitination in centrosome regulation, ciliary basal body-plasma membrane docking, vesicle-mediated transport, and signaling (Figure 4A). In the MF-related GO term category 24 terms were significantly enriched. Of these, the 8 most enriched MF terms (Fisher’s exact test value ≤5.5E-3) indicated that this dataset is most specific for K63-linked polyubiquitination. All other enriched functions were involved in protein trafficking and signaling, and included ‘phospholipid binding’, ‘signaling receptor binding’/‘receptor ligand activity’, and ‘clathrin binding’. These functions are in line with biological processes pertaining to vesicle transport *via* ESCRT-mediated K63 ubiquitination-linked endocytosis of signaling receptors (Figure 4B). Finally, in the last category, 46 CC GO terms were significantly overrepresented. The 15 most enriched terms (Fisher’s exact test value ≤7.5E-4) were again mostly linked to either regulation of processes occurring at the basal body/centriolar satellites or clathrin-coated vesicles (Figure 4C).

Altogether, the UBD proximity labeling approach yielded a highly specific dataset indicating the important and novel role of K63-linked polyubiquitination in regulating ciliary function in IMCD3 cells *via* the ESCRT-dependent clathrin-mediated endocytosis pathway.

### Ubiquitinome of RPE1 cilia indicates a critical role of caveolae

**FIGURE 5:**
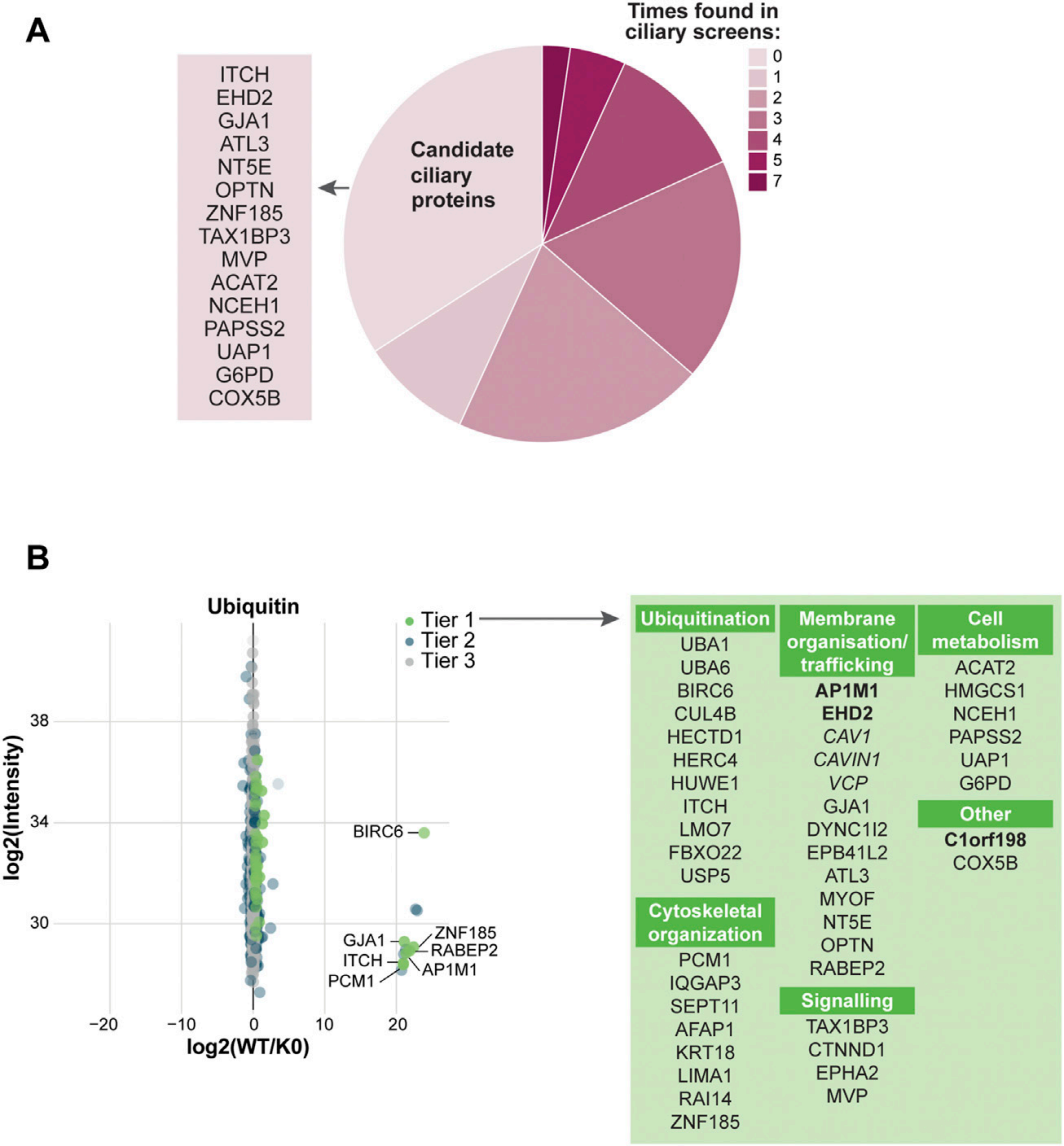
Analysis of UAP mass spectrometry results **(A)** Comparison of the significantly enriched proteins with our ciliary database. Most proteins were previously identified in other ciliary screens, of which PCM1, VCP, and DYNC1I2 were identified most often (dark pink). The remaining proteins are indicated as ‘Candidate ciliary proteins’ on the left **(B)** MS analysis of the enriched proteins for the WT ubiquitin *versus* the K0 ubiquitin variant. Proteins are colored according to their significance tier and all Tier 1 significant proteins were grouped by function (green). The proteins related to clathrin-mediated endocytosis (bold) and non-clathrin-mediated endocytosis (italic) are marked. The complete list of identified proteins can be found in Supplementary Table S2.

To expand the ciliary ubiquitinome dataset, the UAP approach was developed in RPE1 cells. Similar to the UBD-PL approach, 6 replicates per cell line were subjected to mass spectrometry analysis following HA-based immunoprecipitation. This yielded a total of 1272 proteins that were present in at least 4 of the 6 WT samples. Statistical analysis revealed a total of 44 proteins that were Tier 1 enriched (q-value ≤0.05 & Sign. A ≤ 0.05), and additional 22 proteins that were enriched in Tier 2 (*p*-value ≤0.05 & SignA ≤0.05) (Supplementary Table S2). Comparing the significantly enriched proteins to published cilia-specific datasets revealed that 66% of the proteins identified in our UAP-based approach have previously been linked to cilia. Among these proteins was PCM1, which was also identified in our UBD screen (Figure 5A).

Since ubiquitin itself was used as a bait for this approach, by far the largest group of proteins identified with this method included proteins related to the ubiquitin cascade. This comprised E1 enzymes (UBA1, UBA6), the deubiquitinating enzyme USP5, and a number of E3 ligases (BIRC6, CUL4B, HERC4, HUWE1, HECTD1, ITCH, FBXO22, and LMO7). UBA1 and UBA6 have previously been found in the cilia proteome of IMCD3 cells (Mick et al., 2015). A variety of targets involved in the tightly interconnected groups of proteins involved in cytoskeletal organization and membrane trafficking was identified (Mick et al., 2015), such as: AFAP1, LIMA1, RAI14, ZNF185, EPB41L2, AP1M1, MYOF, RABEP2, and GJA1. Fewer proteins were identified in the categories of signaling and cell metabolism. Interestingly, three out of six the proteins identified in the latter category are involved in cholesterol metabolism (ACAT2, HMGCS1 and NCEH1). The most striking group of proteins identified in this screen, however, were several main structural components of caveolae, flask-shaped cholesterol-enriched invaginations on the cell membrane (Ariotti and Parton 2013; Parton and del Pozo 2013). These proteins were namely CAV1, CAVIN1 (PTRF) and EHD2 (Figure 5B). Moreover, two proteins with known involvement in CAV1 ubiquitination were also present in our screen, VCP (Tier 1) and ANKRD13A (Tier 2) (Hayer et al., 2010; Kirchner et al., 2013; Burana et al., 2016).

**FIGURE 6:**
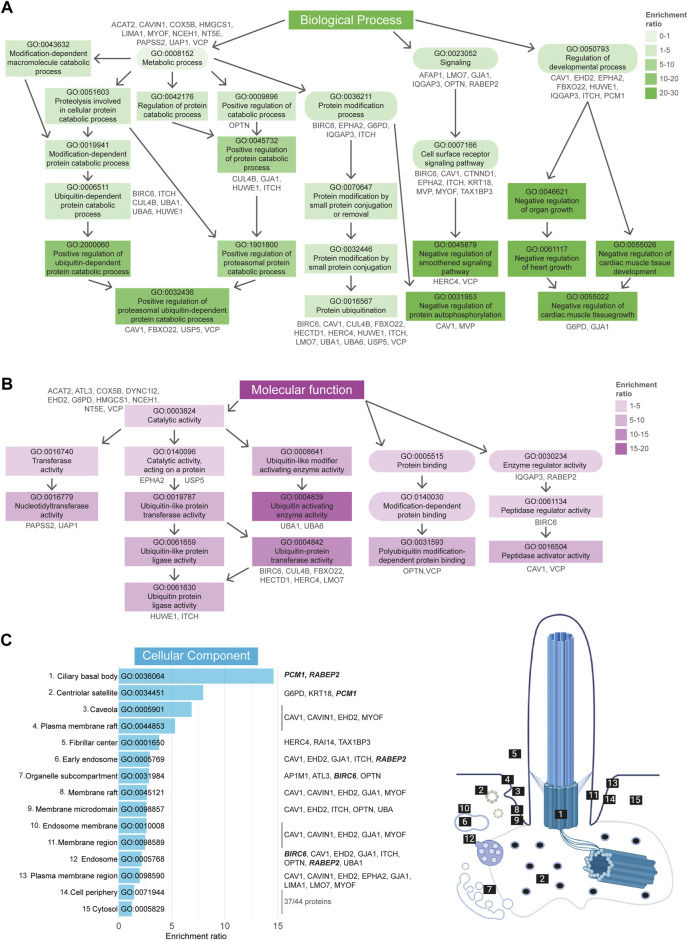
Gene Ontology enrichment analysis of UAP dataset. **(A)** GO term enrichment for biological processes (BP) colored by their enrichment ratio. The 19 most enriched terms (Fisher’s exact test value ≤1.5E-3) are displayed (square) along with supporting GO terms (circle). Arrows indicate the associated terms and the genes per term are indicated in grey. All upstream terms also include the genes from the related downstream terms. **(B)** GO term enrichment analysis for molecular functions (MF) for which all 13 significantly enriched MF terms (Fisher’s exact test value ≤0.05) are displayed as in **(A)**. **(C)** All 15 significantly enriched terms (Fisher’s exact test value ≤0.05) of the cellular components (CC) GO term enrichment analysis are displayed, ordered by their enrichment ratio. The cellular position of these terms and their related proteins are indicated on the right. The term “Fibrillar center” refers to the nuclear region involved in transcription of ribosomal RNA, however the genes associated here with this term were also shown to localize to the cilia (dashed line). The same applied for the terms “Cell periphery” and “Cytosol”, for which the related genes were not indicated, as there were too many to clearly display. Proteins indicated in bold italic letters were shared hits between UBD-PL and UAP. Non-etheless, a complete list of proteins can be found in Supplementary Table S2.

GO term enrichment analysis was performed as described in the previous section. GO BP analysis revealed a total of 180 enriched terms, of which the 50 most enriched terms are indicated in Supplementary Figure S2. Of these, the 19 most enriched terms (Fisher’s exact test value ≤1.5E-3) can be divided into three groups, “Metabolic process”, “Signaling”, and “Regulation of developmental process” (Figure 6A). GO term MF enrichment analysis yielded a total 13 significantly enriched MF terms (Fisher’s exact test value ≤0.05), most of which pertained to functions involved in the ubiquitination cascade (Figure 6B). The last enrichment analysis regarding CC GO terms resulted in a total of 15 significantly enriched terms (Fisher’s exact test value ≤0.05). The most enriched terms in this category were related to the ciliary basal body, centriolar satellites, and caveolae (Figure 6C).

In conclusion, our UAP-based approach in RPE1 cells identified potential novel key players in cilia-specific ubiquitination and more specifically, the regulation of CAV1 in the context of caveolae *via* ubiquitination.

### DOMINO active subnetwork identification analysis

**FIGURE 7:**
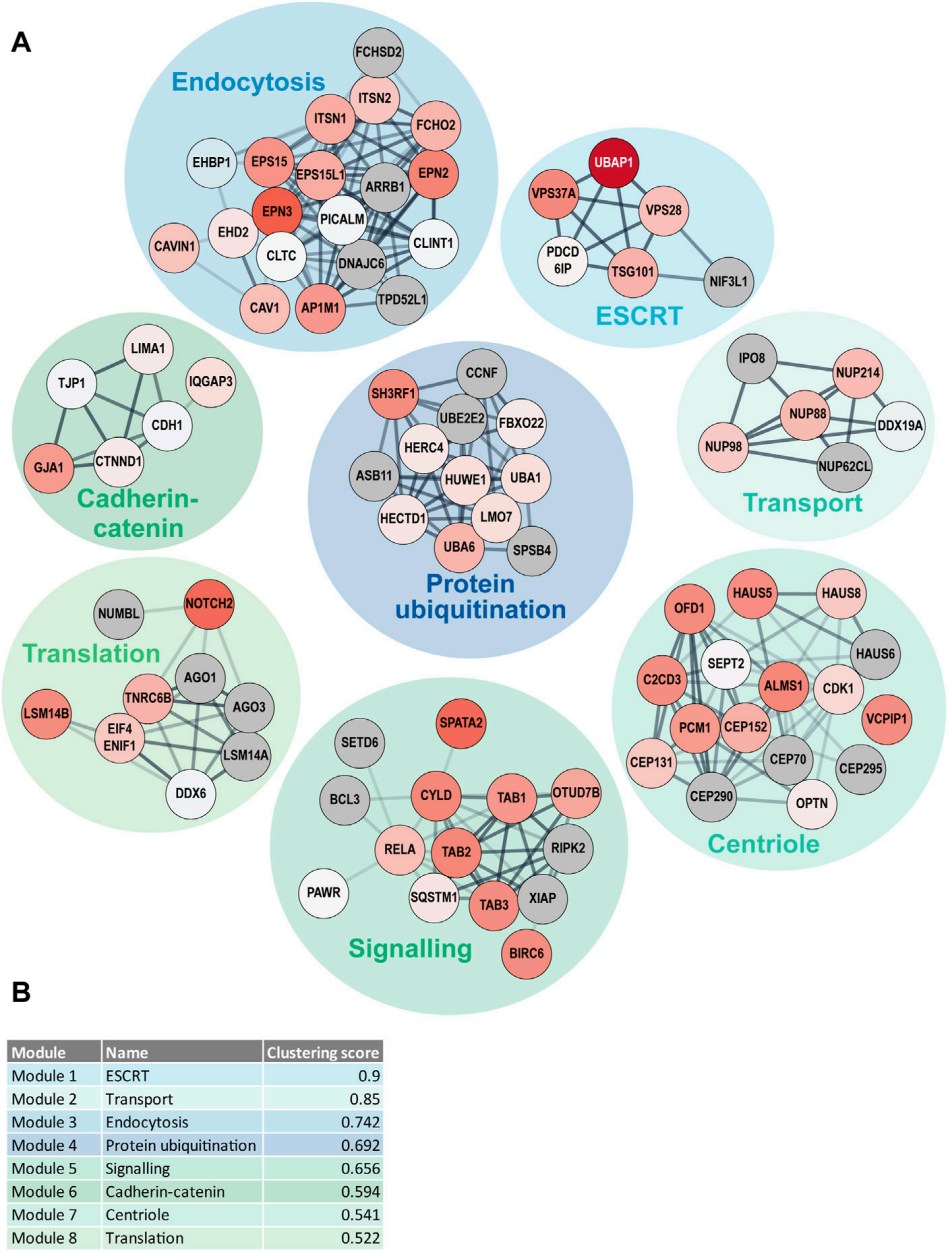
DOMINO active network identification **(A)** DOMINO was used to identify active subnetworks in the combined data from the UBD-PL and UAP approaches. The node colors indicate the enrichment ratios from depleted (blue) to highly enriched (dark red) proteins. Grey nodes indicate proteins that were not detected in either of our approaches. The edge thickness was determined by the STRING confidence score and the edge transparency displays the edge betweenness. **(B)** The modules were ranked in accordance with their clustering score. Module names indicate the most enriched GO terms. A full list of all enriched GO-terms per module can be found in Supplementary Table S3.

In addition to the GO term enrichment analysis, DOMINO active network identification was performed. For this analysis, the combined UBD-PL and UAP data were used in order to identify the modules of the complete network that are involved in and regulated by ciliary ubiquitination (Figure 7). Despite the use of only Tier 1 proteins for the DOMINO analysis, the identified modules also contained many of the Tier 2 and Tier 3 proteins. This results in an overview of mainly enriched, but also some depleted proteins in the modules, highlighting the internal regulations within each module. Most significant are the ESCRT, transport, and endocytosis modules. The latter also includes the caveolae proteins CAV1, CAVIN1, and EHD2. A list of the GO terms enriched in each of the modules can be found in Supplementary Table S3.

### Dynamic ciliary localization of CAV1

**FIGURE 8:**
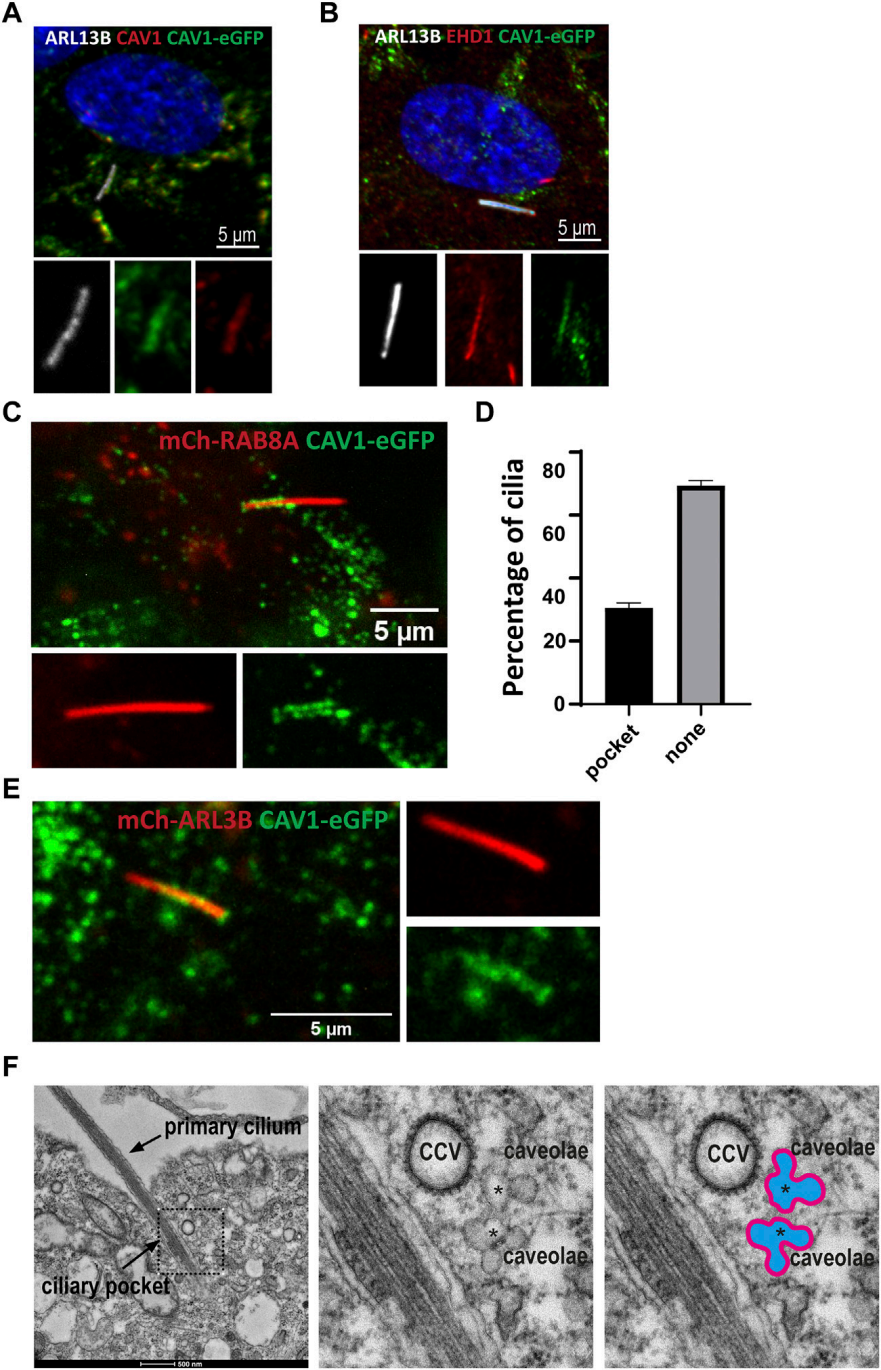
Dynamic CAV1 localization at the ciliary pocket **(A)** A stable RPE1Flp-In cell line expressing eGFP-tagged CAV1 was generated for live cell imaging purposes. The recombinant CAV1 (green) colocalizes with both endogenous CAV1 stained with an anti-CAV1 antibody (red) and the ciliary marker ARL13B (white) **(B)** The ciliary pocket marker EHD1 (red) colocalizes with CAV1 (green) and the ciliary protein ARL13B (white). **(C)** RPE1 CAV1-eGFP cells stably expressing RAB8A-mCherry underwent live imaging (5–20 min) showing dynamic accumulation of CAV1-eGFP at the cilia pocket. This is a single time frame representation **(D)** Quantification of CAV1-eGFP ciliary localization from time frame images as in C (n = 75). **(E)** Single time frame of RPE1 CAV1-eGFP cells stably expressing ARL13B-mCherry shows similar cilia localization as in **(C)**. **(F)** Representative TEM micrograph of a primary cilium in RPE1 cells serum-starved for 48 h and processed for TEM. Clusters of interconnected caveolae (asterisks) and clathrin coated vesicles (CCV) are found close to the ciliary pocket and the base of the primary cilium.

Several previous studies have documented the presence of caveolae or CAV1 at the cilium-centrosome axis in different contexts, and have demonstrated important roles for CAV1 in regulating ciliary length and signaling (Schou et al., 2017; Rangel et al., 2019). To better understand the role of caveolae and ubiquitination of CAV1 in ciliary function, localization experiments were carried out in RPE1 cells. Although not present in all instances, in co-localization experiments using antibodies against CAV1 (Supplementary Figure S3A) or CAVIN1 (Supplementary Figure S3B) together with the ciliary membrane marker ARL13B, the two caveolae proteins were detected in the proximal ciliary region suggestive of TZ and CiPo localization (Supplementary Figure S3), in agreement with previous work (Schou et al., 2017; Pedersen et al., 2016). To confirm this observation, co-staining was performed using an antibody against EHD1 as a marker for the CiPo compartment (Lu et al., 2015). In RPE1 Flp-In cells stably expressing CAV1 fused C-terminally to the eGFP fluorescent tag, CAV1 co-localized with EHD1 in the CiPo, when both proteins were present in this compartment (Figure 8B). Native expression of the recombinant CAV1 protein was confirmed *via* a co-staining using an antibody against endogenous CAV1 and ARL13B was used to visualize the ciliary compartment (Figure 8A).

Next, we sought to better characterize the localization of CAV1 by means of live imaging microscopy. Using lentiviral transduction, mCherry-RAB8A or mCherry-ARL13B were stably introduced into the RPE1 CAV1-eGFP Flp-In cell line to mark the cilia. Tracking the localization of CAV1 over time (5–20 min) revealed a dynamic distribution of the protein at the CiPo (30% of total cilia, n = 75) and the base of cilia (Figures 8C, D; Supplementary Video S1). To confirm that this effect is not a consequence of mCherry-RAB8A expression, we performed identical experiments where mCherry-ARL13B was expressed as a cilia marker instead. CAV1-eGFP exhibited the dynamic pocket localization observed in RAB8A-expressing cells (Figure 8E, Supplementary Video S2). Similar observations were made when cilia were labeled with the microtubule dye SiR-Tubulin (Supplementary Figure S4). When comparing the SiR-Tubulin images to those of mCherry-RAB8A/ARL13B, it became evident that the basal CAV1-eGFP localization that we observed directly distal to ARL13B or RAB8A is not the basal body (labelled by the SiR-Tubulin dye), but very likely corresponds to the TZ (Supplementary Figure S4) (Schou et al., 2017).

Finally, the presence of caveolae at the CiPo was interrogated be means of transmission electron microscopy (TEM). Clusters of caveolae and clathrin coated vesicles (CCV) were readily detected close to the CiPo and the base of the primary cilium (Figure 8F) (Ariotti and Parton 2013; Parton and del Pozo 2013; Pedersen et al., 2016). To confirm that the rosette-like structures found in conventional TEM micrographs are indeed caveolae, we specifically stained caveolae using an RPE1 cell line stably expressing CAVIN1-APEX2-eGFP (Supplementary Figure S3C). Confocal microscopy analysis showed that CAVIN1 localized to the CiPo compartment (Supplementary Figure S3D), as expected. APEX2 staining followed by TEM revealed clusters of caveolae closely associated with the CiPo and the ciliary base (Supplementary Figures S3E–I).

### Ubiquitin-mediated turnover of CAV1 at the ciliary pocket regulates ciliary length

**FIGURE 9:**
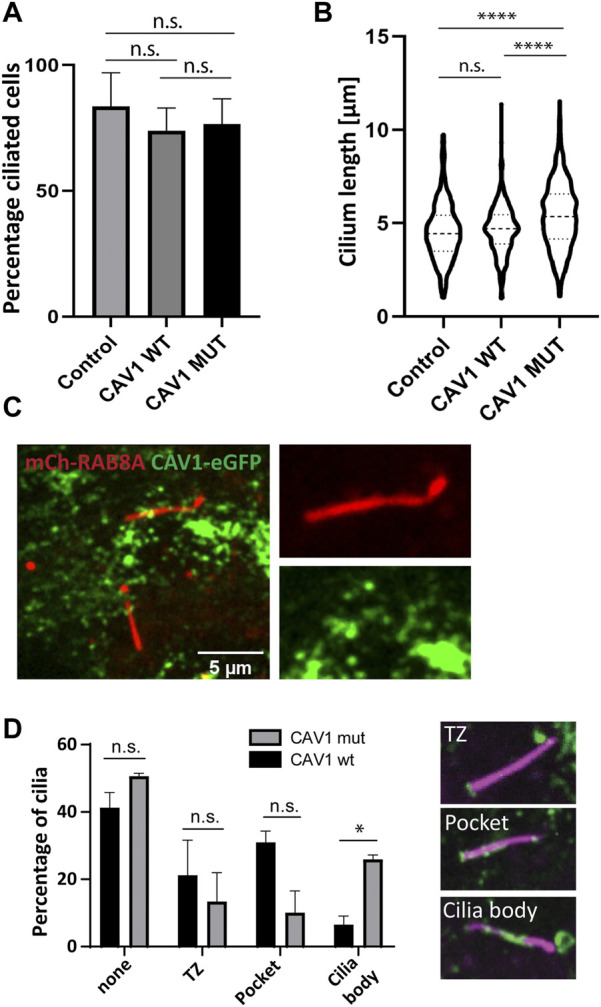
Ciliary length but not ciliation affected in CAV1 mutant lines. Three biological replicates and three technical replicates were imaged in order to determine ciliation levels and ciliary length in RPE1 Flp-In cells stably expressing eGFP-tagged CAV1 wild-type (CAV1 WT), or a CAV1 ubiquitination-impaired mutant (CAV1 MUT), and an untransfected control (Control). ARL13B was used as a marker for the ciliary length. The ALPACA tool was used to quantify both parameters. **(A)** The levels of ciliation between the three lines did not differ significantly. The percentage of ciliated cells was as follows: Control - 83%, CAV1 WT - 73%, and CAV1 MUT - 76%. Error bars indicate mean with SD. **(B)** A significant difference (*p* < 0.0001) in cilia length was observed between the CAV1 MUT (cilia counted = 589), as compared to both the CAV1 WT (cilia counted = 422) and Control lines (cilia counted = 341). The latter did not differ from each other significantly. **(C)** A single time frame from live cell imaging of RPE1 CAV1-eGFP MUT cells stably expressing RAB8A-mCherry. **(D)** Comparison of CAV1-eGFP WT and CAV-eGFP MUT cilia localization imaged as in **(C)**. Total of 78 (WT) or 77 (MUT) cilia were imaged and quantified. Localization was classified as shown: none for no cilia association, TZ for transition zone only localization, Pocket localization includes the transition zone and the pocket, and Body for lump-like localization on the ciliary body (as seen in C). Significance based on *p* < 0.05.

The presence of VCP (Tier 1), a known ciliogenesis regulator (Raman et al., 2015), and ANKRD13A (Tier 2) in our ciliary UAP screen pointed to the specific contribution of CAV1 ubiquitination in the regulation of its ciliary localization and function (Kirchner et al., 2013; Burana et al., 2016). To study this, a ubiquitination-impaired CAV1 Flp-In cell line model was established. A CAV1 lysine-less mutant, which has been previously described (Kirchner et al., 2013), was fused C-terminally to eGFP and stably expressed in RPE1 Flp-In cells. The ALPACA (Accumulation and Length Phenotype Automated Cilia Analysis) (Doornbos et al., 2021) tool was used to compare ciliation levels, as well as cilia length, in RPE1 cells stably expressing either the wild type or mutant CAV1 fusion protein. To verify that the stably transfected wild type CAV1 does not itself affect these variables, as an additional control, the parental untransfected RPE1 Flp-In cell line was used. ARL13B was used as a ciliary marker. While the percentage of ciliated cells was not affected across the three cell lines (Figure 9A), cilia length was significantly longer (*p* < 0.0001) in the CAV1 mutant cells compared to both the CAV1 wild type, as well as the parental RPE1 Flp-In cell line (Figure 9B). Since previous work showed that CAV1 depletion similar causes ciliary lengthening (Schou et al., 2017; Rangel et al., 2019) these results suggests that the ubiquitination-impaired CAV1 mutant functions as a dominant negative.

We next wondered whether changes in the localization of the ubiquitination-impaired CAV1 mutant protein were the cause for this ciliary lengthening phenotype. As described in the previous section, mCherry-RAB8A was stably expressed in addition to the mutant CAV1-eGFP in RPE1 Flp-In cells, to serve as a marker for cilia (Figure 9C). Live cell imaging demonstrated that mutant CAV1 was still able to localize at the CiPo, however the protein accumulated in lumps associated with the proximal region of the cilium. These were rarely observed in the case of the wild type CAV1 protein (Figure 9D; Supplementary Video S3). This accumulation could indicate inefficient turnover of the mutant CAV1 protein at the CiPo, and a role for timely CAV1 ubiquitination in the regulation of ciliary length.

## Discussion

Once considered a vestigial organelle, the cilium is now being recognized as an integral player in the regulation of cellular processes and the maintenance of cellular homeostasis. Dysregulation in ciliary functions results in multi-organ deficiencies, but the precise mechanisms underlying this dysfunction remain poorly understood. In 2015, Datta et al. showed that accumulation of non-outer segment proteins in photoreceptor cells ultimately caused photoreceptor degeneration in the classical ciliopathy Bardet-Biedl Syndrome (Datta et al., 2015). Later, Shinde et al. demonstrated that defects in a gene causative for BBS, resulted in the abnormal accumulation of ubiquitinated proteins in the photoreceptor outer segments of affected mice (Shinde et al., 2020). Interestingly, similar phenotypes caused by aberrant ubiquitin-mediated degradation, have long been a hallmark of neurodegenerative diseases such as Parkinson’s disease and Alzheimer’s disease, and therapeutic approaches to target these are being developed (Schmidt et al., 2021). These parallels and the avenues of therapeutic possibilities they could offer to patients suffering from ciliopathies should be examined. However, our knowledge of the contribution of ubiquitination in ciliary function and dysfunction is rather fragmentary.

To address this gap, our study aimed at developing unbiased proteomics-based approaches to categorize the processes that are regulated by ubiquitination in the primary cilia: a first molecular blueprint of the ciliary ubiquitinome. This may serve as a guide to understand the breadth of modes in which this particular group of posttranslational modifications can regulate ciliary signaling and proteostasis, unravelling new facets of ciliary biology in health and disease. While the two approaches that we developed successfully achieved their goal, they both have particular strengths and limitations that warrant discussion. Since a canonical ciliary targeting signal, if it exists, is yet to be identified, this study made use of a previously published sequence in order to target ubiquitin or ubiquitin-binding domains into the cilium (Mick et al., 2015; Kohli et al., 2017; May et al., 2021a). While effective, the N-terminal region of NPHP3 used in this and other studies, still retains a large portion of the NPHP3 protein (aa 1–203), which could be a limiting factor in detecting a broader range of interacting proteins. A potential solution to that could be the addition of a longer flexible linker between the NPHP3-targeting signal and the bait proteins, or exploring other ciliary-targeting options. Due to its small size and versatility, ubiquitin is not ideally suited for tagging purposes. We chose to explore polyubiquitination which is conducted *via* the C-terminal Gly residue of ubiquitin, leaving linear chain formation–a modification requiring the N-terminus of ubiquitin–outside the scope of this study. In addition, ubiquitin binding domains, although used out of the context of their native proteins in this study, could still have binding affinity to specific types of polyubiquitin chains introducing bias in the final results.

Nonetheless, both the UAP and the UBD-PL screens, resulted in the identification of known and novel targets of cilia-specific ubiquitination. A number of these interactions were recorded at the base/centriolar satellite region, which could be explained by the expected variability in stages of ciliation, as indicated by the co-localization of the ciliary-targeted UBD with PCM in the early stages of ciliogenesis (Supplementary Figure S5). Non-etheless, the presence of PCM1, which was highly significant (Tier 1) using both methods, shows the validity of this study in identifying cilia-relevant ubiquitination events. Extensive regulation of PCM1 *via* ubiquitination through a network of proteins including another target identified in our screens, CYLD, has been well described (Wang et al., 2016; Wang P. et al., 2019; Douanne et al., 2019). Comparing the two datasets indicated a significant overlap in the identified processes and their localization, since both methods yielded a high enrichment of proteins involved in signaling and protein trafficking at the base of primary cilia.

While the contribution of ubiquitination to these tightly interconnected processes is well-described in literature (Shearer and Saunders 2016; Anvarian et al., 2019; Nachury and Mick 2019; Shinde et al., 2020), this study provides a comprehensive overview of the specific proteins involved in ubiquitin-dependent regulation of ciliary trafficking and signaling. In addition, it highlights possible mechanistic differences based on distinctive features in the architecture of the cilium. Remarkably, the cilia of RPE1 cells differ vastly in terms of organization of the periciliary membrane, which in these cells is invaginated to form the CiPo, compared to the cilia of IMCD3 cells which by and large lack a CiPo compartment (Molla-Herman et al., 2010; Ghossoub et al., 2011). It would be interesting to explore this not only in regards to ubiquitination in the cilium but also in the context of other ciliary processes. ESCRT-dependent internalization of ubiquitinated membrane receptors *via* the clathrin endocytic pathway has thus far not been implicated as a major mechanism in the regulation of ciliary signal transduction in the kidney. However, this pathway was at the forefront of our UBD-PL dataset obtained in the kidney-derived IMCD3 cell line, raising the question whether dysfunction of clathrin-mediated endocytosis could be involved in renal ciliopathies. Polycystic kidney disease (PKD), a hallmark ciliopathy, is caused by mutations in the PKD1 and PKD2 genes which encode for the polycystin 1 (PC1), and 2- (PC2) proteins respectively. Notably, work performed in *C. elegans* has shown that inactivation of ESCRT-0 components causes the abnormal accumulation of ubiquitinated PC2 at the ciliary base demonstrating the importance of the ESCRT-0 proteins in downregulating the polycystin complex (Hu et al., 2007). Moreover, proteomics analysis of the isolated TZ of Chlamydomonas, revealed the presence of 6 ESCRT proteins (Diener et al., 2015). The TOM1L2 clathrin adaptor protein was in fact found inside the cilia of IMCD3 cells by immunofluorescence (Mick et al., 2015) and has more recently been shown to directly participate in the removal of ubiquitinated cargo from the cilium (Shinde et al., 2022). Alternatively, ubiquitination of target proteins mediated by the ESCRT complex may be a sorting rather than a degradative pathway. Peripherin-2, a protein crucial for photoreceptor ciliary function, follows an ESCRT-0 mediated route for correct ciliary targeting and mutations perturbing this interaction could be causative for retinal degeneration (Otsu et al., 2019). Exit of K63 polyubiquitinated GPCRs from the cilium *via* a β-arrestin-BBSome-dependent mechanism was recently described for Hedgehog and somatostatin signaling (Shinde et al., 2020). It is possible that in light of our UBD-PL data, and as also suggested by the authors of the paper, this complex further links to the lysosomal degradative pathway *via* ESCRT and could further be extended to include Notch, TGF-β and Wnt signaling. An advantage of our dataset is that it also uncovers novel candidate ciliary proteins, such as FAM175B and WRNIP1, both of which have been implicated in the regulation of K63 ubiquitination (Zeqiraj et al., 2015; Tan et al., 2017; Zhao et al., 2019). Interestingly, FAM175B (a.k.a. ABRX2 or KIAA0157) a member of the BRISC complex, is specifically involved in the cytoplasmic retention and microtubule attachment to kinetochores of this DUB complex (Feng et al., 2010). It is thus tempting to speculate a moonlighting role of this protein at the cilium, e.g. in cilium disassembly, similar to many other mitotic regulators (Doornbos and Roepman 2021).

Ubiquitin-mediated signaling *via* non-clathrin caveolar endocystosis, on the other hand, seems to be the predominant mechanism in RPE1 cells. Considering the reported role of clathrin endocytosis at the CiPo of RPE1 cells (Pedersen et al., 2016) and the interconnectivity between clathrin and non-clathrin endocytosis, it is conceivable that mechanistically these pathways act sequentially, providing different layers of fine-tuning in the mediation of signal transduction. Caveolae are cholesterol-rich bulb-shaped membrane invaginations which, through interactions with actin modifiers, PIPs and membrane receptors, are speculated to provide a malleable platform for the regulation of signaling pathways (Parton 2018). It has been demonstrated that CAV1 resides in cholesterol-rich membrane microdomains together with the Hedgehog signaling regulators SMO and PTCH1, and an interaction exists between CAV1 and PTCH1 (Karpen et al., 2001; Yue et al., 2014). In addition, CAV1 localizes to the transition zone of cilia in a KIF13B-dependent manner, where it promotes Hedgehog signaling (Schou et al., 2017). Interestingly, a number of ubiquitin targets identified in the Tier 1 of our UAP screen have been linked to Hedgehog signaling. The HECT E3 ligase ITCH negatively regulates the canonical Hedgehog pathway through ubiquitination of either Numb or SuFu (Infante et al., 2019), and Numb was recently shown to localize to the ciliary pocket of mouse fibroblasts (Liu et al., 2022). Ciliogenesis regulator Rab effector protein, RABEP2, which was highly significant in both screens, localizes to the cilium and connects to Hedgehog *via* the subdistal appendage protein SDCCAG8 (Airik et al., 2016). Moreover, EPB4L2, a cytoskeletal adaptor protein, and the K48-specific deubiquitinase USP5, have both been identified as interactors of SDCCAG8 *via* tandem affinity purification (Boldt et al., 2016). It remains to be determined whether these proteins participate in a complex that orchestrates the ciliary Hedgehog axis and how ubiquitination of one or multiple components contributes to the multifaceted regulation of this pathway.

The presence of both VCP and ANKRD13A provides evidence that in part this mechanism is regulated by ubiquitination of CAV1 itself. The ubiquitin-interacting motif of ANKRD13A binds preferentially to K63-modified CAV1 oligomers and cooperates with VCP to facilitate trafficking of CAV1 to the lysosomal compartment (Burana et al., 2016). Perturbing CAV1 ubiquitination, similarly to siRNA-mediated knock down of CAV1 (Rangel et al., 2019; Schou et al., 2017) resulted in the elongation of cilia beyond their normal size, suggesting a dominant negative effect. Based on our live cell imaging data, it is highly plausible that this phenotype is caused by decreased turnover of CAV1 at the periciliary membrane. Although mutations in CAV1 are mostly associated with a lipodystrophy phenotype, it is worth noting that Berger et al. (Berger et al., 2002) reported a patient with atypical lipodystrophy who also suffered from retinitis pigmentosa, and later Cao et al. (Cao et al., 2008) established a CAV1 truncating mutation as the causative genetic aberration. It is possible that the dynamic distribution of CAV1 at the ciliary base will affect its contribution to ciliopathy-like phenotypes. It would therefore be of utmost interest to determine the ciliary length of CAV1 patient-derived fibroblasts and investigate potential ubiquitin-mediated protein turnover defects.

The exact mechanism through which CAV1 ubiquitination regulates ciliary length would be an interesting avenue for future investigation. Caveolae are highly enriched for specific lipid modifications, such as PI(4,5)P_2_ (Fujita et al., 2009). In fact, ubiquitination of lysine residues in the PI(4,5)P2-binding region of CAVIN1 serves as a sensor for membrane association and dissociation (Zhou et al., 2021). Recent work from Stilling et al. (Stilling et al., 2022) demonstrated that PI(4,5)P_2_ levels are strictly regulated in the cilium and determine ciliary length. PI(4,5)P_2_ was more concentrated at the ciliary base and depleted towards the tip, and depletion of ciliary PI(4,5)P_2_ led to longer cilia. In light of this, determining the potential decrease of PI(4,5)P_2_ in CAV1 ubiquitination-deficient cells would be of significant interest.

In conclusion, our combined proteomics approaches provide a rich resource for future studies to unravel ubiquitin-regulated mechanisms that contribute to maintaining ciliary proteostasis. Our demonstration of the localization of caveolae at the ciliary pocket of RPE1 cells and the ubiquitination of CAV1 that specifically plays a role in the regulation of ciliary length emphasizes the versatility of this dataset.

## Data Availability

The original contributions presented in the study are included in the article/Supplementary Material, further inquiries can be directed to the corresponding author.
