# Supplementary material for: A targeted multi-proteomics approach generates a blueprint of the ciliary ubiquitinome
Source: Front Cell Dev Biol. 2023 Jan 26;11:1113656. doi: 10.3389/fcell.2023.1113656 (PMC9908615; doi:10.3389/fcell.2023.1113656)
Supplement: Supplementary file 6 [file DataSheet1.DOCX]

**Supplemental tables and figures**

**A targeted multi-proteomics approach generates a blueprint of the ciliary ubiquitinome**

Mariam. G. Aslanyan^1*^, Cenna Doornbos^1*^, Gaurav D. Diwan^2,5†^, Zeinab Anvarian^3†^, Tina Beyer^4^, Katrin Junger^4^, Sylvia E.C. van Beersum^1^, Robert B. Russell^2,5^, Marius Ueffing^4^, Alexander Ludwig^6^, Karsten Boldt^4^, Lotte B. Pedersen^3^, Ronald Roepman^1±^

*1 Radboud University Medical Center, Department of Human Genetics and Radboud Institute for Molecular Life Sciences, Nijmegen, The Netherlands*

*2 BioQuant, Heidelberg University, Heidelberg, Germany*

*3 University of Copenhagen, Department of Biology, Section for Cell Biology and Physiology, Copenhagen, Denmark*

*4 Eberhard Karl University of Tübingen, Institute for Ophthalmic Research, Tübingen, Germany*

*5 Biochemistry Center (BZH), Heidelberg University, Heidelberg, Germany*

*6 School of Biological Sciences and NTU Institute of Structural Biology, Nanyang Technological University, Singapore City 637551, Singapore*

** these authors share first authorship*

*† these authors contributed equally to this work*

*± Corresponding author:* [*Ronald.Roepman@radboudumc.nl*](mailto:Ronald.Roepman@radboudumc.nl)

# Supplemental tables

****Supplemental Table 1. Full dataset of UBD-enriched proteins****

**List of all Tier 1 and Tier 2 enriched proteins identified by UBD RAD23B-based proximity-labelling as displayed in Figure 1E. Proteins are sorted by significance A score and q-value.**

| **Uniprot ID** | **Protein** | **Median**  **Samples** | **Median**  **Controls** | **p-value** | **q-value** | **LFQ ratio**  **sample/control** | **Significance**  **A score** | **Tier** |
| --- | --- | --- | --- | --- | --- | --- | --- | --- |
| O35551 | RABEP1 | 23.91 | 0.00 | 1.09E-13 | 1.80E-10 | 2.39E+01 | 0.00E+00 | Tier 1 |
| P54728 | RAD23B | 31.72 | 0.00 | 1.05E-12 | 4.32E-10 | 3.17E+01 | 0.00E+00 | Tier 1 |
| Q8BMK0 | CEP85 | 26.29 | 0.00 | 9.53E-13 | 4.32E-10 | 2.63E+01 | 0.00E+00 | Tier 1 |
| O88746 | TOM1 | 25.18 | 0.00 | 8.43E-13 | 4.32E-10 | 2.52E+01 | 0.00E+00 | Tier 1 |
| Q01705; Q61982 | NOTCH1 | 26.50 | 0.00 | 3.39E-12 | 7.00E-10 | 2.65E+01 | 0.00E+00 | Tier 1 |
| Q8CHU3 | EPN2 | 26.10 | 0.00 | 2.46E-12 | 7.00E-10 | 2.61E+01 | 0.00E+00 | Tier 1 |
| Q8CHS8 | VPS37A | 25.05 | 0.00 | 2.88E-12 | 7.00E-10 | 2.50E+01 | 0.00E+00 | Tier 1 |
| Q80WC7 | AGFG2 | 24.06 | 0.00 | 3.18E-12 | 7.00E-10 | 2.41E+01 | 0.00E+00 | Tier 1 |
| Q7TS75 | AMER1 | 25.49 | 0.00 | 4.06E-12 | 7.45E-10 | 2.55E+01 | 0.00E+00 | Tier 1 |
| O55126 | GBAS | 24.87 | 0.00 | 5.78E-12 | 9.53E-10 | 2.49E+01 | 0.00E+00 | Tier 1 |
| Q9JLG8 | CAPN15 | 26.77 | 0.00 | 7.54E-12 | 1.13E-09 | 2.68E+01 | 0.00E+00 | Tier 1 |
| Q69ZI1 | SH3RF1 | 24.18 | 0.00 | 1.13E-11 | 1.55E-09 | 2.42E+01 | 0.00E+00 | Tier 1 |
| Q6A037 | N4BP1 | 23.58 | 0.00 | 1.39E-11 | 1.76E-09 | 2.36E+01 | 0.00E+00 | Tier 1 |
| Q7TN98; Q812E0; Q7TN99 | CPEB4; CPEB2; CPEB3 | 23.97 | 0.00 | 2.54E-11 | 3.00E-09 | 2.40E+01 | 0.00E+00 | Tier 1 |
| Q8K2J4 | CCDC14 | 26.88 | 25.44 | 2.25E-07 | 2.47E-05 | 1.44E+00 | 7.32E-03 | Tier 1 |
| Q60902 | EPS15L1 | 29.08 | 27.36 | 3.57E-07 | 3.68E-05 | 1.73E+00 | 1.66E-03 | Tier 1 |
| Q9Z0R4 | ITSN1 | 28.74 | 27.02 | 5.86E-07 | 5.69E-05 | 1.72E+00 | 1.76E-03 | Tier 1 |
| Q9R0L6 | PCM1 | 30.58 | 28.31 | 8.01E-07 | 7.34E-05 | 2.28E+00 | 5.18E-05 | Tier 1 |
| Q7M6Y3; Q61548 | PICALM | 27.93 | 26.70 | 1.55E-06 | 1.30E-04 | 1.23E+00 | 1.83E-02 | Tier 1 |
| O35516 | NOTCH2 | 28.82 | 25.62 | 1.90E-06 | 1.44E-04 | 3.20E+00 | 2.37E-08 | Tier 1 |
| Q64337 | SQSTM1 | 28.44 | 26.19 | 1.91E-06 | 1.44E-04 | 2.25E+00 | 6.21E-05 | Tier 1 |
| P42567 | EPS15 | 27.33 | 25.08 | 2.37E-06 | 1.70E-04 | 2.25E+00 | 6.37E-05 | Tier 1 |
| Q8BKI2 | TNRC6B | 29.33 | 27.77 | 4.44E-06 | 2.93E-04 | 1.56E+00 | 4.02E-03 | Tier 1 |
| Q8CF89 | TAB1 | 25.88 | 23.72 | 5.53E-06 | 3.38E-04 | 2.16E+00 | 1.20E-04 | Tier 1 |
| Q6PFD9 | NUP98 | 26.17 | 25.18 | 7.23E-06 | 4.11E-04 | 9.94E-01 | 4.58E-02 | Tier 1 |
| P46662 | NF2 | 28.11 | 25.13 | 8.34E-06 | 4.58E-04 | 2.98E+00 | 1.89E-07 | Tier 1 |
| Q8VE88 | FAM114A2 | 28.12 | 27.01 | 1.02E-05 | 5.43E-04 | 1.11E+00 | 2.98E-02 | Tier 1 |
| Q8C6E0 | CFAP36 | 26.61 | 25.32 | 1.16E-05 | 5.78E-04 | 1.28E+00 | 1.45E-02 | Tier 1 |
| Q99L00 | HAUS8 | 27.42 | 26.36 | 1.15E-05 | 5.78E-04 | 1.06E+00 | 3.65E-02 | Tier 1 |
| P33215 | NEDD1 | 27.78 | 26.44 | 1.39E-05 | 6.54E-04 | 1.35E+00 | 1.11E-02 | Tier 1 |
| Q61187 | TSG101 | 26.63 | 25.04 | 1.82E-05 | 7.70E-04 | 1.59E+00 | 3.33E-03 | Tier 1 |
| Q80TQ2 | CYLD | 27.58 | 25.08 | 2.85E-05 | 1.02E-03 | 2.51E+00 | 9.81E-06 | Tier 1 |
| Q8BZI0 | AFAP1L1 | 26.62 | 24.68 | 2.94E-05 | 1.03E-03 | 1.94E+00 | 4.90E-04 | Tier 1 |
| Q9EST3 | EIF4ENIF1 | 27.18 | 26.11 | 3.34E-05 | 1.15E-03 | 1.07E+00 | 3.45E-02 | Tier 1 |
| G3X9J0 | SIPA1L3 | 29.78 | 28.76 | 3.52E-05 | 1.18E-03 | 1.03E+00 | 4.04E-02 | Tier 1 |
| Q99KN9 | CLINT1 | 29.66 | 28.51 | 4.08E-05 | 1.32E-03 | 1.14E+00 | 2.59E-02 | Tier 1 |
| Q91VS8 | FARP2 | 28.24 | 27.20 | 4.16E-05 | 1.32E-03 | 1.04E+00 | 3.84E-02 | Tier 1 |
| Q9D1C8 | VPS28 | 26.95 | 25.69 | 5.95E-05 | 1.71E-03 | 1.25E+00 | 1.67E-02 | Tier 1 |
| Q62036 | CEP131 | 30.82 | 29.73 | 5.94E-05 | 1.71E-03 | 1.09E+00 | 3.25E-02 | Tier 1 |
| Q9CZ44 | NSFL1C | 28.21 | 26.72 | 8.72E-05 | 2.21E-03 | 1.49E+00 | 5.58E-03 | Tier 1 |
| A2AUM9 | CEP152 | 25.60 | 23.96 | 1.17E-04 | 2.83E-03 | 1.64E+00 | 2.69E-03 | Tier 1 |
| Q8C886 | PLEKHN1 | 26.87 | 25.46 | 1.33E-04 | 3.18E-03 | 1.41E+00 | 8.19E-03 | Tier 1 |
| E9PYK3 | PARP4 | 29.58 | 27.18 | 1.88E-04 | 4.03E-03 | 2.40E+00 | 2.11E-05 | Tier 1 |
| Q8CEC0 | NUP88 | 26.78 | 25.41 | 1.98E-04 | 4.20E-03 | 1.37E+00 | 9.98E-03 | Tier 1 |
| Q80U93 | NUP214 | 28.09 | 26.75 | 2.19E-04 | 4.40E-03 | 1.34E+00 | 1.14E-02 | Tier 1 |
| Q8C7U1 | N4BP3 | 28.99 | 27.90 | 2.17E-04 | 4.40E-03 | 1.09E+00 | 3.25E-02 | Tier 1 |
| Q8CDG3 | VCPIP1 | 27.58 | 25.19 | 2.59E-04 | 5.09E-03 | 2.39E+00 | 2.28E-05 | Tier 1 |
| Q04207 | RELA | 25.30 | 24.01 | 2.71E-04 | 5.23E-03 | 1.29E+00 | 1.40E-02 | Tier 1 |
| Q9QXJ1 | APBB1 | 26.43 | 25.35 | 3.83E-04 | 6.95E-03 | 1.07E+00 | 3.44E-02 | Tier 1 |
| Q91W69; Q80VP1 | EPN3 | 27.95 | 24.50 | 5.42E-04 | 8.95E-03 | 3.45E+00 | 1.98E-09 | Tier 1 |
| Q80U22 | RUSC2 | 27.64 | 26.46 | 6.08E-04 | 9.83E-03 | 1.19E+00 | 2.18E-02 | Tier 1 |
| Q9Z0R6 | ITSN2 | 26.73 | 25.60 | 6.66E-04 | 1.06E-02 | 1.12E+00 | 2.83E-02 | Tier 1 |
| O88738 | BIRC6 | 26.08 | 24.53 | 9.39E-04 | 1.37E-02 | 1.55E+00 | 4.21E-03 | Tier 1 |
| Q91VW9 | ZKSCAN3 | 24.57 | 23.45 | 1.22E-03 | 1.67E-02 | 1.12E+00 | 2.86E-02 | Tier 1 |
| Q6NZK5 | KIAA1328 | 23.50 | 0.00 | 1.32E-03 | 1.73E-02 | 2.35E+01 | 0.00E+00 | Tier 1 |
| Q8BLK9 | RPS6KC1 | 26.81 | 24.72 | 1.29E-03 | 1.73E-02 | 2.10E+00 | 1.78E-04 | Tier 1 |
| Q68FH0 | PKP4 | 26.26 | 25.24 | 1.29E-03 | 1.73E-02 | 1.01E+00 | 4.27E-02 | Tier 1 |
| Q99K90 | TAB2 | 25.55 | 0.00 | 1.45E-03 | 1.86E-02 | 2.56E+01 | 0.00E+00 | Tier 1 |
| Q80Z25 | OFD1 | 24.43 | 0.00 | 1.64E-03 | 2.02E-02 | 2.44E+01 | 0.00E+00 | Tier 1 |
| Q91WG2 | RABEP2 | 24.43 | 0.00 | 1.68E-03 | 2.02E-02 | 2.44E+01 | 0.00E+00 | Tier 1 |
| Q3TCJ1 | FAM175B | 23.77 | 0.00 | 1.77E-03 | 2.10E-02 | 2.38E+01 | 0.00E+00 | Tier 1 |
| P59281 | ARHGAP39 | 23.49 | 0.00 | 1.84E-03 | 2.15E-02 | 2.35E+01 | 0.00E+00 | Tier 1 |
| Q8BJS8 | MTBP | 22.67 | 0.00 | 1.87E-03 | 2.16E-02 | 2.27E+01 | 0.00E+00 | Tier 1 |
| Q91XU0 | WRNIP1 | 24.55 | 0.00 | 2.05E-03 | 2.26E-02 | 2.45E+01 | 0.00E+00 | Tier 1 |
| Q921I6 | SH3BP4 | 24.05 | 0.00 | 2.05E-03 | 2.26E-02 | 2.40E+01 | 0.00E+00 | Tier 1 |
| Q571K4 | TAB3 | 23.99 | 0.00 | 2.05E-03 | 2.26E-02 | 2.40E+01 | 0.00E+00 | Tier 1 |
| P50136 | BCKDHA | 23.95 | 0.00 | 2.05E-03 | 2.26E-02 | 2.40E+01 | 0.00E+00 | Tier 1 |
| Q9D786 | HAUS5 | 23.74 | 0.00 | 2.06E-03 | 2.26E-02 | 2.37E+01 | 0.00E+00 | Tier 1 |
| Q52KB6 | C2CD3 | 23.56 | 0.00 | 2.05E-03 | 2.26E-02 | 2.36E+01 | 0.00E+00 | Tier 1 |
| Q9QWV9 | CCNT1 | 23.42 | 0.00 | 2.13E-03 | 2.32E-02 | 2.34E+01 | 0.00E+00 | Tier 1 |
| Q8K4E0 | ALMS1 | 25.37 | 0.00 | 7.49E-03 | 6.37E-02 | 2.54E+01 | 0.00E+00 | Tier 2 |
| P51612 | XPC | 25.55 | 0.00 | 8.85E-03 | 7.19E-02 | 2.56E+01 | 0.00E+00 | Tier 2 |
| Q5SRX1 | TOM1L2 | 25.23 | 0.00 | 8.96E-03 | 7.25E-02 | 2.52E+01 | 0.00E+00 | Tier 2 |
| O88811 | STAM2 | 25.87 | 0.00 | 1.06E-02 | 8.25E-02 | 2.59E+01 | 0.00E+00 | Tier 2 |
| E9Q309 | CEP350 | 25.20 | 0.00 | 1.06E-02 | 8.25E-02 | 2.52E+01 | 0.00E+00 | Tier 2 |
| Q9DBG3; O35643 | AP2B1 | 24.25 | 0.00 | 1.10E-02 | 8.45E-02 | 2.42E+01 | 0.00E+00 | Tier 2 |
| O08807 | PRDX4 | 27.23 | 0.00 | 1.25E-02 | 8.74E-02 | 2.72E+01 | 0.00E+00 | Tier 2 |
| Q9R060 | NUBP1 | 24.45 | 0.00 | 1.16E-02 | 8.74E-02 | 2.44E+01 | 0.00E+00 | Tier 2 |
| Q8BTS4 | NUP54 | 24.14 | 0.00 | 1.25E-02 | 8.74E-02 | 2.41E+01 | 0.00E+00 | Tier 2 |
| Q8R4R6 | NUP35 | 24.00 | 0.00 | 1.19E-02 | 8.74E-02 | 2.40E+01 | 0.00E+00 | Tier 2 |
| Q91Z49 | FYTTD1 | 23.96 | 0.00 | 1.26E-02 | 8.74E-02 | 2.40E+01 | 0.00E+00 | Tier 2 |
| Q9D1H8 | MRPL53 | 23.95 | 0.00 | 1.25E-02 | 8.74E-02 | 2.40E+01 | 0.00E+00 | Tier 2 |
| Q9D0N7 | CHAF1B | 23.73 | 0.00 | 1.21E-02 | 8.74E-02 | 2.37E+01 | 0.00E+00 | Tier 2 |
| Q8K3D3 | SWI5 | 23.57 | 0.00 | 1.25E-02 | 8.74E-02 | 2.36E+01 | 0.00E+00 | Tier 2 |
| Q6ZPG2 | WDR90 | 23.46 | 0.00 | 1.25E-02 | 8.74E-02 | 2.35E+01 | 0.00E+00 | Tier 2 |
| Q8VE47 | UBA5 | 23.42 | 0.00 | 1.25E-02 | 8.74E-02 | 2.34E+01 | 0.00E+00 | Tier 2 |
| Q640Q5 | PAN3 | 23.38 | 0.00 | 1.25E-02 | 8.74E-02 | 2.34E+01 | 0.00E+00 | Tier 2 |
| P52431 | POLD1 | 23.10 | 0.00 | 1.28E-02 | 8.74E-02 | 2.31E+01 | 0.00E+00 | Tier 2 |
| Q9JI78 | NGLY1 | 23.08 | 0.00 | 1.25E-02 | 8.74E-02 | 2.31E+01 | 0.00E+00 | Tier 2 |
| Q9EQW7 | KIF13A | 23.04 | 0.00 | 1.17E-02 | 8.74E-02 | 2.30E+01 | 0.00E+00 | Tier 2 |
| P97429 | ANXA4 | 23.04 | 0.00 | 1.25E-02 | 8.74E-02 | 2.30E+01 | 0.00E+00 | Tier 2 |
| P30285 | CDK4 | 23.02 | 0.00 | 1.22E-02 | 8.74E-02 | 2.30E+01 | 0.00E+00 | Tier 2 |
| Q8K2X3 | OBFC1 | 22.87 | 0.00 | 1.25E-02 | 8.74E-02 | 2.29E+01 | 0.00E+00 | Tier 2 |
| Q9JLI8 | SART3 | 22.06 | 0.00 | 1.27E-02 | 8.74E-02 | 2.21E+01 | 0.00E+00 | Tier 2 |
| Q9CZW4 | ACSL3 | 23.65 | 0.00 | 1.33E-02 | 8.96E-02 | 2.36E+01 | 0.00E+00 | Tier 2 |
| Q8CEE6 | PASK | 26.23 | 23.21 | 2.51E-02 | 1.49E-01 | 3.02E+00 | 1.37E-07 | Tier 2 |
| Q8BH48 | UBAP1 | 27.06 | 21.94 | 2.65E-02 | 1.55E-01 | 5.12E+00 | 1.21E-18 | Tier 2 |
| Q64331 | MYO6 | 25.00 | 21.93 | 2.87E-02 | 1.66E-01 | 3.07E+00 | 8.05E-08 | Tier 2 |
| Q5DU05 | CEP164 | 25.85 | 23.26 | 2.99E-02 | 1.71E-01 | 2.59E+00 | 4.93E-06 | Tier 2 |
| Q61194 | PIK3C2A | 23.83 | 22.01 | 3.21E-02 | 1.80E-01 | 1.82E+00 | 9.70E-04 | Tier 2 |
| Q7TQK4 | EXOSC3 | 24.43 | 22.25 | 3.22E-02 | 1.80E-01 | 2.18E+00 | 1.02E-04 | Tier 2 |
| Q9WVM1 | RACGAP1 | 25.46 | 23.45 | 3.25E-02 | 1.80E-01 | 2.01E+00 | 3.19E-04 | Tier 2 |
| Q8CGF6 | WDR47 | 25.11 | 23.23 | 3.28E-02 | 1.81E-01 | 1.89E+00 | 6.62E-04 | Tier 2 |
| Q922Y1 | UBXN1 | 25.42 | 23.61 | 3.39E-02 | 1.83E-01 | 1.81E+00 | 1.04E-03 | Tier 2 |
| Q9D281 | FAM114A1 | 24.50 | 22.89 | 3.35E-02 | 1.83E-01 | 1.61E+00 | 3.00E-03 | Tier 2 |
| Q8VCW8 | ACSF2 | 23.95 | 22.58 | 3.39E-02 | 1.83E-01 | 1.36E+00 | 1.02E-02 | Tier 2 |
| Q9JK48 | SH3GLB1 | 24.47 | 0.00 | 3.43E-02 | 1.83E-01 | 2.45E+01 | 0.00E+00 | Tier 2 |
| Q3UIW5 | RNF10 | 24.24 | 0.00 | 3.45E-02 | 1.83E-01 | 2.42E+01 | 0.00E+00 | Tier 2 |
| Q8VBT9 | ASPSCR1 | 24.05 | 0.00 | 3.53E-02 | 1.83E-01 | 2.41E+01 | 0.00E+00 | Tier 2 |
| O88413 | TULP3 | 23.75 | 0.00 | 3.53E-02 | 1.83E-01 | 2.38E+01 | 0.00E+00 | Tier 2 |
| P46737 | BRCC3 | 23.60 | 0.00 | 3.51E-02 | 1.83E-01 | 2.36E+01 | 0.00E+00 | Tier 2 |
| Q8VCH8 | UBXN4 | 23.32 | 0.00 | 3.45E-02 | 1.83E-01 | 2.33E+01 | 0.00E+00 | Tier 2 |
| A2ADY9; Q9DAF3 | DDI2 | 29.91 | 25.32 | 3.47E-02 | 1.83E-01 | 4.58E+00 | 2.62E-15 | Tier 2 |
| Q8VC30 | DAK | 23.88 | 22.30 | 3.41E-02 | 1.83E-01 | 1.59E+00 | 3.46E-03 | Tier 2 |
| Q61425 | HADH | 23.68 | 22.17 | 3.44E-02 | 1.83E-01 | 1.50E+00 | 5.24E-03 | Tier 2 |
| Q8VDF2 | UHRF1 | 24.09 | 22.60 | 3.49E-02 | 1.83E-01 | 1.49E+00 | 5.50E-03 | Tier 2 |
| P25799 | NFKB1 | 23.91 | 0.00 | 3.61E-02 | 1.83E-01 | 2.39E+01 | 0.00E+00 | Tier 2 |
| Q9D1P0 | MRPL13 | 23.76 | 0.00 | 3.63E-02 | 1.83E-01 | 2.38E+01 | 0.00E+00 | Tier 2 |
| P70271 | PDLIM4 | 25.63 | 24.47 | 3.61E-02 | 1.83E-01 | 1.16E+00 | 2.47E-02 | Tier 2 |
| Q8C5L3 | CNOT2 | 24.00 | 0.00 | 3.65E-02 | 1.84E-01 | 2.40E+01 | 0.00E+00 | Tier 2 |
| Q8K4Z5 | SF3A1 | 24.04 | 0.00 | 3.72E-02 | 1.85E-01 | 2.40E+01 | 0.00E+00 | Tier 2 |
| Q8CIG8 | PRMT5 | 23.61 | 0.00 | 3.72E-02 | 1.85E-01 | 2.36E+01 | 0.00E+00 | Tier 2 |
| P97305 | NFATC3 | 24.17 | 22.86 | 3.73E-02 | 1.85E-01 | 1.30E+00 | 1.34E-02 | Tier 2 |
| Q4VGL6 | RC3H1 | 24.65 | 0.00 | 3.86E-02 | 1.88E-01 | 2.46E+01 | 0.00E+00 | Tier 2 |
| Q8BYA0 | TBCD | 24.30 | 0.00 | 3.87E-02 | 1.88E-01 | 2.43E+01 | 0.00E+00 | Tier 2 |
| Q9D0K2 | OXCT1 | 23.51 | 0.00 | 3.84E-02 | 1.88E-01 | 2.35E+01 | 0.00E+00 | Tier 2 |
| Q9QZK2 | BCAR3 | 23.94 | 22.75 | 3.83E-02 | 1.88E-01 | 1.19E+00 | 2.20E-02 | Tier 2 |
| Q62087 | PON3 | 23.12 | 0.00 | 4.00E-02 | 1.93E-01 | 2.31E+01 | 0.00E+00 | Tier 2 |
| Q8BIE6 | FRMD4A | 23.11 | 0.00 | 4.02E-02 | 1.94E-01 | 2.31E+01 | 0.00E+00 | Tier 2 |

****Supplemental Table 2. Full dataset of UAP-enriched proteins****

**Tier 1 and Tier 2 significant proteins that were identified by ubiquitin-based affinity proteomics, as displayed in Figure 1E. Proteins are sorted by significance A score and q-value.**

| **Uniprot ID** | **Protein** | **Median**  **Samples** | **Median**  **Controls** | **p-value** | **q-value** | **LFQ ratio**  **sample/control** | **Significance**  **A score** | **Tier** |
| --- | --- | --- | --- | --- | --- | --- | --- | --- |
| O15231 | ZNF185 | 22.39 | 0.00 | 7.12E-33 | 9.05E-30 | 2.24E+01 | 0.00E+00 | Tier 1 |
| Q15154 | PCM1 | 20.96 | 0.00 | 1.30E-12 | 8.30E-10 | 2.10E+01 | 0.00E+00 | Tier 1 |
| Q9H5N1 | RABEP2 | 21.86 | 0.00 | 4.02E-12 | 1.65E-09 | 2.19E+01 | 0.00E+00 | Tier 1 |
| Q6NZI2 | PTRF | 27.48 | 26.32 | 5.19E-12 | 1.65E-09 | 1.16E+00 | 2.82E-11 | Tier 1 |
| Q03135; P56539 | CAV1 | 25.45 | 24.17 | 8.69E-11 | 2.21E-08 | 1.28E+00 | 2.75E-13 | Tier 1 |
| P45974 | USP5 | 26.19 | 24.92 | 1.10E-10 | 2.33E-08 | 1.27E+00 | 4.19E-13 | Tier 1 |
| P22314 | UBA1 | 27.61 | 27.05 | 5.72E-10 | 1.04E-07 | 5.62E-01 | 7.57E-04 | Tier 1 |
| A0AVT1 | UBA6 | 25.24 | 23.73 | 5.47E-09 | 8.69E-07 | 1.52E+00 | 6.77E-18 | Tier 1 |
| Q8WWI1 | LMO7 | 25.69 | 25.08 | 5.52E-08 | 7.81E-06 | 6.05E-01 | 3.20E-04 | Tier 1 |
| Q96J02; Q9H0M0 | ITCH | 20.95 | 0.00 | 6.77E-08 | 8.61E-06 | 2.10E+01 | 0.00E+00 | Tier 1 |
| Q86VI3 | IQGAP3 | 24.32 | 23.73 | 1.03E-07 | 1.19E-05 | 5.92E-01 | 4.22E-04 | Tier 1 |
| Q9NR09; Q96C03 | BIRC6 | 23.87 | 0.00 | 1.63E-07 | 1.73E-05 | 2.39E+01 | 0.00E+00 | Tier 1 |
| O43491 | EPB41L2 | 24.30 | 23.40 | 2.97E-07 | 2.70E-05 | 8.96E-01 | 2.25E-07 | Tier 1 |
| P55072 | VCP | 27.58 | 27.16 | 5.75E-07 | 4.57E-05 | 4.16E-01 | 9.40E-03 | Tier 1 |
| Q9P0K7 | RAI14 | 23.86 | 23.39 | 7.38E-07 | 5.52E-05 | 4.70E-01 | 3.97E-03 | Tier 1 |
| P11413 | G6PD | 26.83 | 26.52 | 7.85E-07 | 5.55E-05 | 3.11E-01 | 3.91E-02 | Tier 1 |
| Q8NEZ5 | FBXO22 | 27.00 | 26.67 | 9.00E-07 | 5.72E-05 | 3.37E-01 | 2.81E-02 | Tier 1 |
| Q6DD88 | ATL3 | 23.68 | 23.37 | 8.73E-07 | 5.72E-05 | 3.08E-01 | 4.04E-02 | Tier 1 |
| P05783; Q497I4 | KRT18 | 27.43 | 27.09 | 1.58E-06 | 9.14E-05 | 3.39E-01 | 2.75E-02 | Tier 1 |
| Q8N556 | AFAP1 | 22.33 | 21.49 | 2.06E-06 | 1.09E-04 | 8.37E-01 | 1.20E-06 | Tier 1 |
| Q96CV9 | OPTN | 21.95 | 21.62 | 4.42E-06 | 2.16E-04 | 3.32E-01 | 3.00E-02 | Tier 1 |
| O14907 | TAX1BP3 | 23.52 | 23.23 | 4.26E-06 | 2.16E-04 | 2.96E-01 | 4.69E-02 | Tier 1 |
| O60716 | CTNND1 | 24.74 | 24.41 | 9.45E-06 | 4.14E-04 | 3.31E-01 | 3.05E-02 | Tier 1 |
| Q7Z6Z7 | HUWE1 | 28.40 | 27.83 | 1.26E-05 | 5.36E-04 | 5.67E-01 | 6.92E-04 | Tier 1 |
| Q13409 | DYNC1I2 | 24.23 | 23.87 | 1.63E-05 | 6.70E-04 | 3.68E-01 | 1.87E-02 | Tier 1 |
| Q9ULT8 | HECTD1 | 21.71 | 21.27 | 3.04E-05 | 1.14E-03 | 4.44E-01 | 6.08E-03 | Tier 1 |
| Q5GLZ8 | HERC4 | 22.70 | 22.31 | 2.98E-05 | 1.14E-03 | 3.89E-01 | 1.39E-02 | Tier 1 |
| Q6PIU2 | NCEH1 | 24.25 | 23.81 | 3.35E-05 | 1.22E-03 | 4.39E-01 | 6.51E-03 | Tier 1 |
| Q9NZM1 | MYOF | 27.62 | 27.25 | 5.46E-05 | 1.83E-03 | 3.61E-01 | 2.05E-02 | Tier 1 |
| Q14764 | MVP | 26.72 | 26.42 | 8.97E-05 | 2.78E-03 | 3.06E-01 | 4.16E-02 | Tier 1 |
| P29317 | EPHA2 | 24.70 | 24.39 | 1.06E-04 | 3.13E-03 | 3.17E-01 | 3.61E-02 | Tier 1 |
| Q16222 | UAP1 | 23.51 | 23.16 | 2.54E-04 | 6.91E-03 | 3.57E-01 | 2.17E-02 | Tier 1 |
| O95340 | PAPSS2 | 24.98 | 24.64 | 2.93E-04 | 7.60E-03 | 3.32E-01 | 3.02E-02 | Tier 1 |
| Q9BWD1 | ACAT2 | 23.90 | 23.50 | 4.73E-04 | 1.12E-02 | 3.99E-01 | 1.20E-02 | Tier 1 |
| P21589 | NT5E | 24.60 | 24.19 | 5.18E-04 | 1.20E-02 | 4.06E-01 | 1.08E-02 | Tier 1 |
| Q9H425 | C1orf198 | 23.44 | 23.10 | 5.70E-04 | 1.29E-02 | 3.41E-01 | 2.68E-02 | Tier 1 |
| P10606 | COX5B | 23.30 | 22.78 | 7.25E-04 | 1.54E-02 | 5.27E-01 | 1.45E-03 | Tier 1 |
| Q13620 | CUL4B | 26.28 | 25.98 | 8.22E-04 | 1.71E-02 | 3.06E-01 | 4.13E-02 | Tier 1 |
| Q01581; P54868 | HMGCS1 | 24.00 | 23.55 | 1.12E-03 | 2.26E-02 | 4.50E-01 | 5.47E-03 | Tier 1 |
| Q9UHB6 | LIMA1 | 23.15 | 22.81 | 1.65E-03 | 3.10E-02 | 3.38E-01 | 2.78E-02 | Tier 1 |
| Q9NVA2; Q14141 | SEPT11 | 24.46 | 24.12 | 1.93E-03 | 3.55E-02 | 3.33E-01 | 2.97E-02 | Tier 1 |
| Q9NZN4 | EHD2 | 25.21 | 24.75 | 2.08E-03 | 3.79E-02 | 4.55E-01 | 5.04E-03 | Tier 1 |
| Q9BXS5; Q9Y6Q5 | AP1M1 | 21.35 | 0.00 | 2.12E-03 | 3.80E-02 | 2.14E+01 | 0.00E+00 | Tier 1 |
| P17302 | GJA1 | 21.10 | 0.00 | 2.21E-03 | 3.90E-02 | 2.11E+01 | 0.00E+00 | Tier 1 |
| P52292 | KPNA2 | 22.75 | 21.88 | 3.23E-03 | 5.48E-02 | 8.77E-01 | 3.90E-07 | Tier 2 |
| P02511 | CRYAB | 22.45 | 20.03 | 3.41E-03 | 5.63E-02 | 2.42E+00 | 1.65E-42 | Tier 2 |
| P46013 | MKI67 | 23.16 | 20.41 | 4.10E-03 | 6.60E-02 | 2.75E+00 | 3.18E-54 | Tier 2 |
| Q8IZ07 | ANKRD13A | 21.66 | 20.53 | 4.43E-03 | 6.82E-02 | 1.13E+00 | 1.09E-10 | Tier 2 |
| P43686 | PSMC4 | 24.31 | 24.00 | 6.57E-03 | 9.09E-02 | 3.13E-01 | 3.82E-02 | Tier 2 |
| Q12983 | BNIP3 | 19.78 | 18.87 | 7.06E-03 | 9.66E-02 | 9.10E-01 | 1.45E-07 | Tier 2 |
| P46060 | RANGAP1 | 23.47 | 23.12 | 7.62E-03 | 1.02E-01 | 3.51E-01 | 2.36E-02 | Tier 2 |
| P30049 | ATP5D | 22.90 | 0.00 | 1.11E-02 | 1.43E-01 | 2.29E+01 | 0.00E+00 | Tier 2 |
| Q8N0X7 | SPG20 | 21.30 | 0.00 | 1.55E-02 | 1.86E-01 | 2.13E+01 | 0.00E+00 | Tier 2 |
| P60953 | CDC42 | 24.63 | 24.33 | 1.70E-02 | 1.90E-01 | 2.98E-01 | 4.55E-02 | Tier 2 |
| Q12846 | STX4 | 21.02 | 20.53 | 1.76E-02 | 1.93E-01 | 4.82E-01 | 3.25E-03 | Tier 2 |
| Q9Y6G9 | DYNC1LI1 | 21.37 | 20.97 | 1.88E-02 | 1.99E-01 | 3.96E-01 | 1.26E-02 | Tier 2 |
| Q9NR30 | DDX21 | 23.35 | 22.68 | 2.35E-02 | 2.32E-01 | 6.68E-01 | 8.20E-05 | Tier 2 |
| Q13085; O00763 | ACACA | 21.84 | 21.23 | 2.40E-02 | 2.34E-01 | 6.05E-01 | 3.22E-04 | Tier 2 |
| Q86W92 | PPFIBP1 | 21.01 | 0.00 | 2.69E-02 | 2.57E-01 | 2.10E+01 | 0.00E+00 | Tier 2 |
| P53367 | ARFIP1 | 20.90 | 20.57 | 2.78E-02 | 2.62E-01 | 3.34E-01 | 2.92E-02 | Tier 2 |
| P49773 | HINT1 | 23.94 | 23.57 | 3.41E-02 | 3.08E-01 | 3.69E-01 | 1.83E-02 | Tier 2 |
| Q9Y696 | CLIC4 | 27.15 | 26.59 | 3.76E-02 | 3.28E-01 | 5.63E-01 | 7.47E-04 | Tier 2 |
| Q9NRX4 | PHPT1 | 23.35 | 22.86 | 4.21E-02 | 3.39E-01 | 4.92E-01 | 2.73E-03 | Tier 2 |
| P20674 | COX5A | 22.68 | 0.00 | 4.32E-02 | 3.41E-01 | 2.27E+01 | 0.00E+00 | Tier 2 |
| Q8WXH0 | SYNE2 | 24.94 | 23.94 | 4.43E-02 | 3.46E-01 | 1.01E+00 | 6.79E-09 | Tier 2 |
| Q9P2R7 | SUCLA2 | 21.11 | 20.80 | 4.97E-02 | 3.76E-01 | 3.15E-01 | 3.71E-02 | Tier 2 |

## **Supplemental figures**


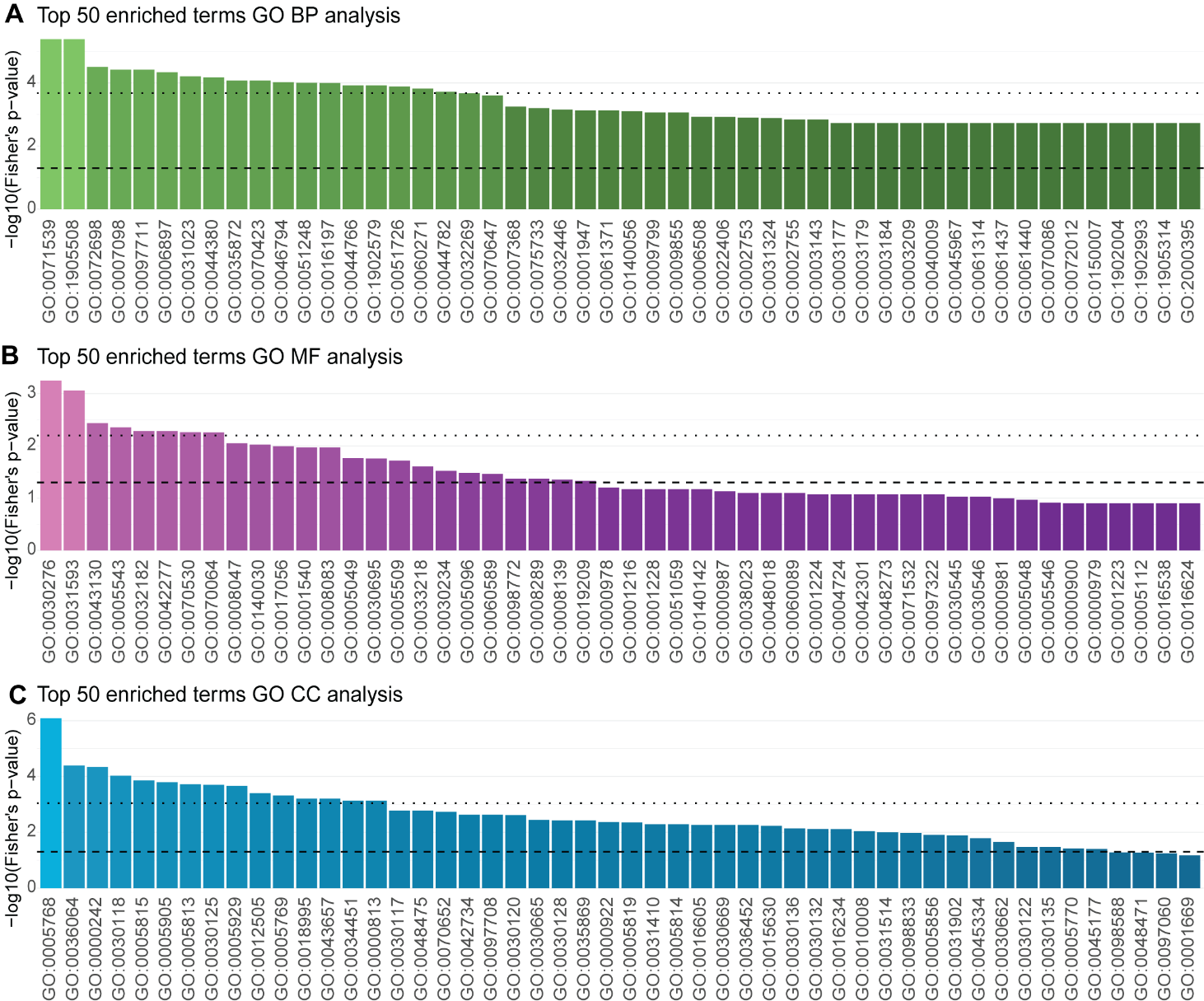


****Supplemental Figure 1. Pathway enrichment analysis of UBD-PL****

**A-C) Top 50 most enriched p GO terms per analysis. Color indications: GO BP analysis in green, GO MF analysis in purple, and GO CC analysis in blue. The dotted line indicates the significance cut-off for the terms included in Figure 3. The dashed line indicates which terms were significantly enriched Fisher’s exact test value ≤ 0.05. Mind that in figure A not all significant terms are displayed because of their abundance.**

## **
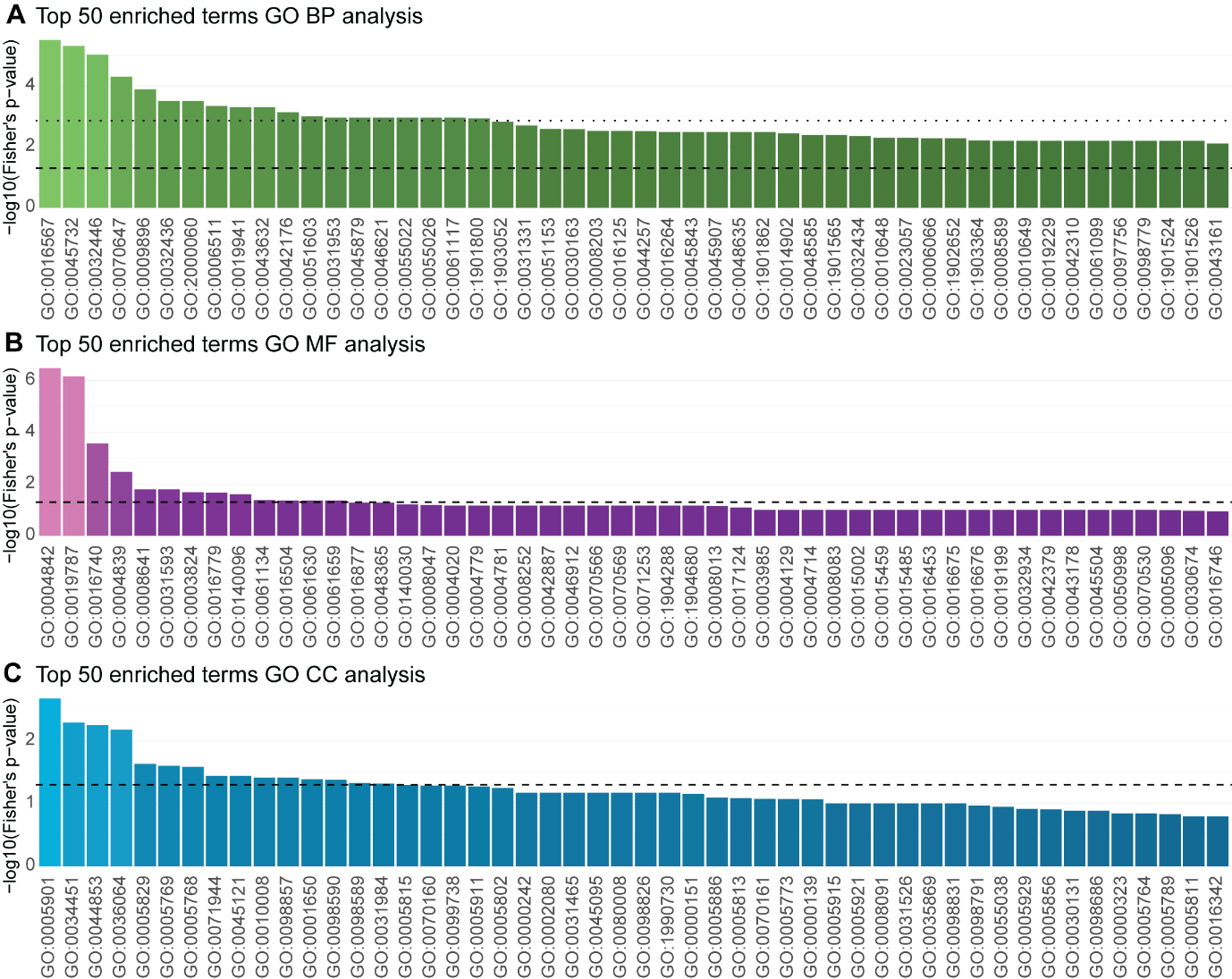
**

Supplemental Figure 2. Pathway enrichment HA pull down

**A-C) GO term enrichment analysis displaying the top 50 results with the most significant terms on the left. Color indications: GO BP analysis in green, GO MF analysis in purple, and GO CC analysis in blue. The dotted line in A indicates the significance cut-off for the terms included in Figure 5. For the others the general significance cut-off was applied based on the adjusted Fisher’s exact test value ≤ 0.05 (dashed line). It has to be noted that in figure A not all significant terms are displayed because of their abundance.**

**
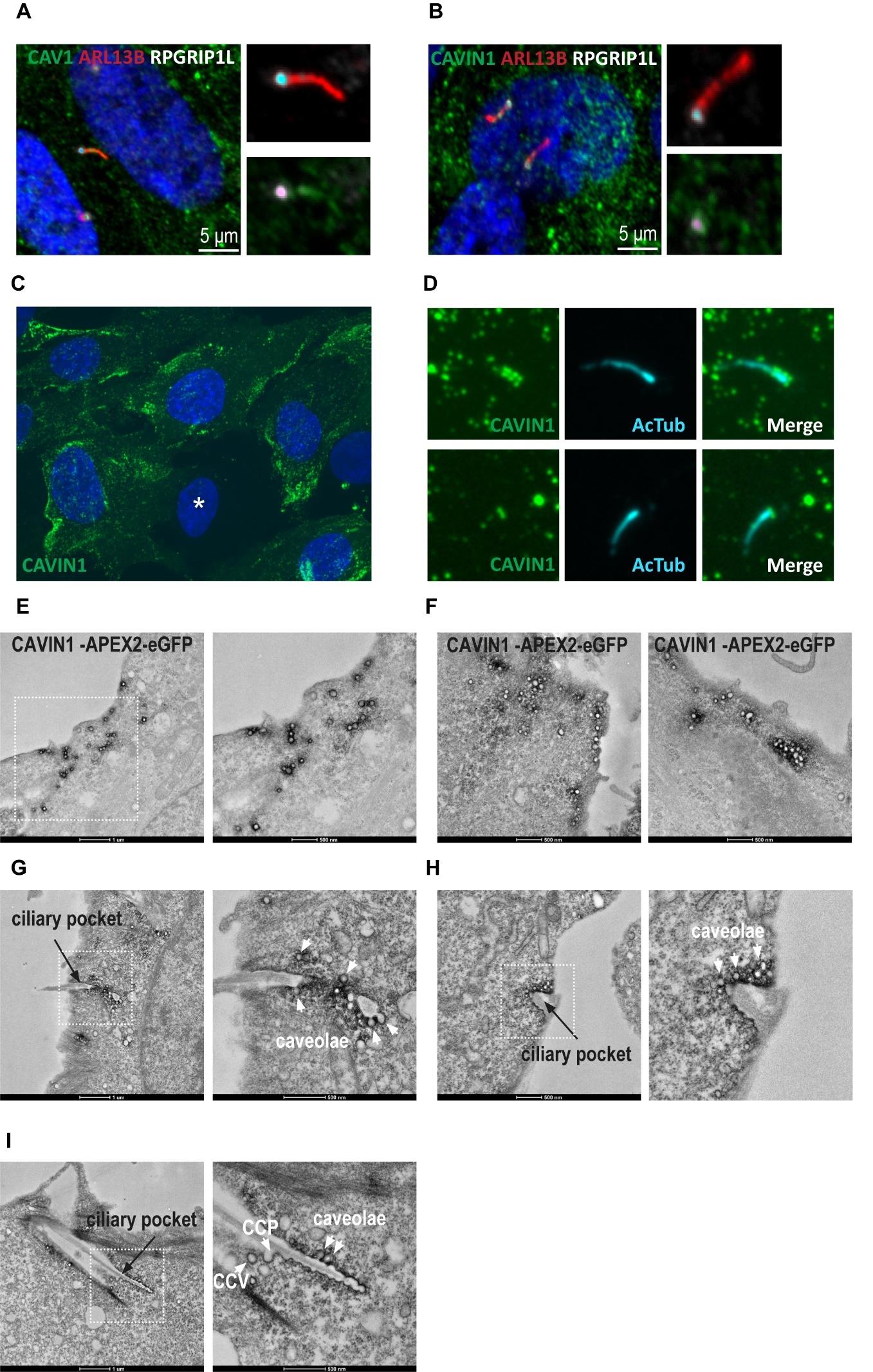
**

**Supplemental Figure 3. Localization of caveolae proteins in RPE1 cells**

**A** and **B)** IF analysis of native CAV1 and CAVIN1 proteins. Endogenous CAV1 (green) and CAVIN1 (green) localization in RPE1 cells was tested using antibodies against the respective proteins. ARL13B (red) was used as marker for cilia, while RPGRIP1L (white) probed the ciliary transition zone (TZ). Both proteins could be detected in the proximal region of the cilium, as well as the TZ. **C)** Localisation of CAVIN1-APEX2-eGFP stably expressed in RPE1 cells. Cells were fixed and counterstained with DAPI and imaged by confocal microscopy. Asterisk indicates an untransfected cell. **D)** The CAVIN1-APEX2-eGFP RPE1 line was serum-starved for 48 hrs, fixed and counterstained with an antibody against acetylated tubulin, and imaged by confocal microscopy. The recombinant CAVIN1-APEX2-eGFP protein was associated with the CiPo (top) and the ciliary base (bottom). **E** and **F)** Representative TEM micrographs of RPE1 cells stably expressing CAVIN1-APEX2-eGFP. Cells were serum-starved, fixed and stained with the APEX2 method. Note the specific staining of caveolae. **G-I)** Representative TEM micrographs of primary cilia in CAVIN1-APEX2-eGFP RPE1 cells. Caveolae are associated with the CiPo and with apparent endocytic structures at the ciliary base.

**
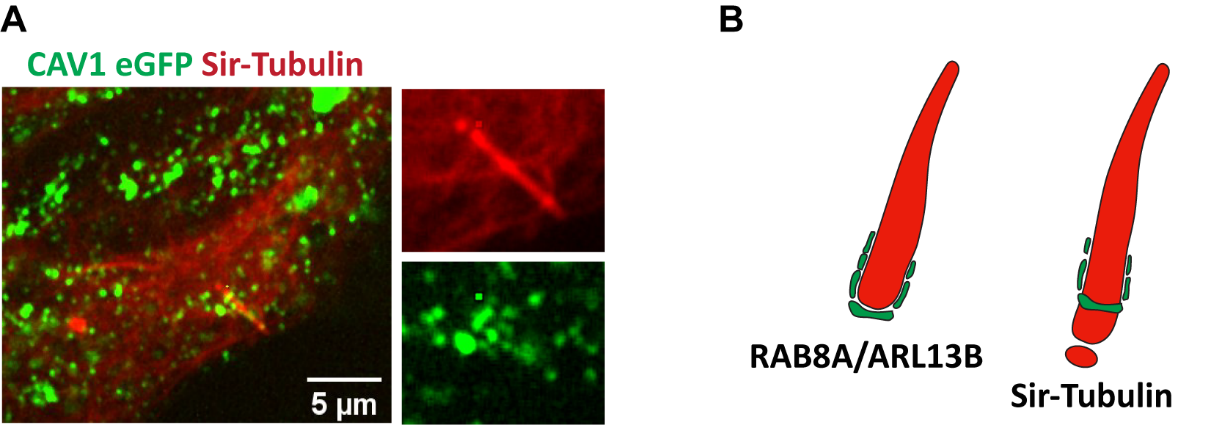
**

**Supplemental Figure 4. CAV1 localizes dynamically in the region of the primary cilium**

**A)** RPE1 CAV1-eGFP cells labelled live with Sir-Tubulin-670 (red) to mark the primary cilium. CAV1-eGFP seems to localize to the TZ and the CiPo. **B)** A schematic showing how CAV1-eGFP is positioned with respect to different cilia markers.


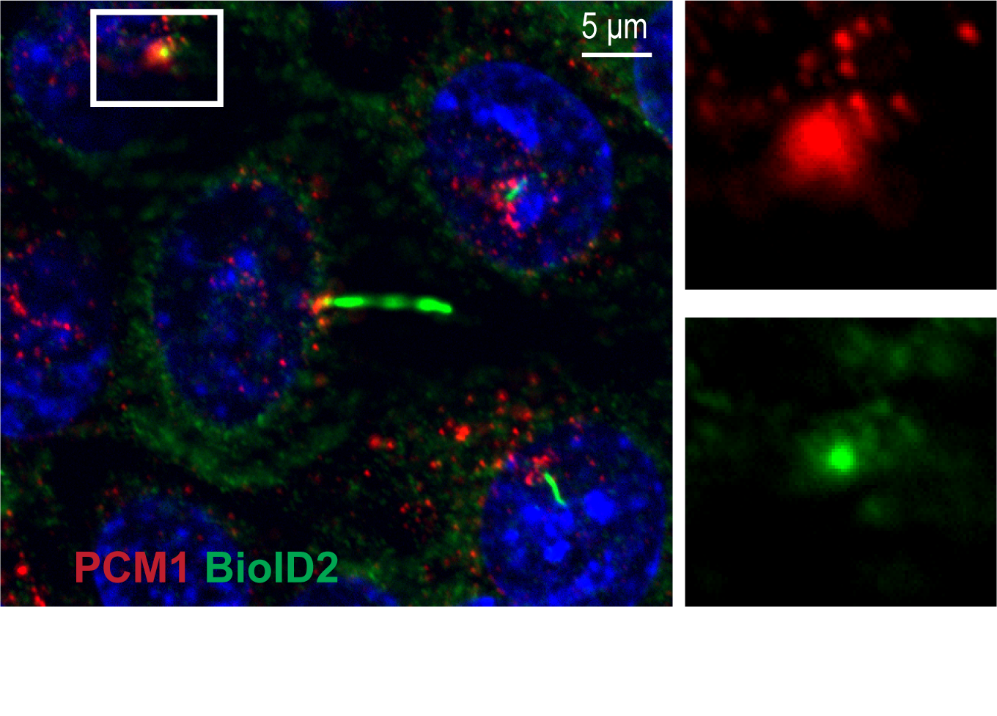


**Supplemental Figure 5. Co-localization of UBD with the centriolar satellite marker PCM1**

IF microscopy analysis of the UBD fused to BioID2 (green) in stable IMCD3 Flp-In cells shows co-localization with the centriolar satellite marker PCM1 (red) in different stages of ciliation. White rectangle indicates a cell in the early stages of ciliogenesis.

**Supplemental videos**

Supplemental video 1

RPE1 CAV1-eGFP wild type cells stably expressing RAB8a-mCherry. Time lapse live cell imaging of cells serum-starved in phenol-free DMEM imaged with spinning disc confocal microscopes with lasers 488 and 561 with 10s time intervals.

Supplemental video 2

RPE1 CAV1-eGFP wild type cells stably expressing ARL13B-mCherry. Time lapse live cell imaging of cells serum-starved in phenol-free DMEM imaged with spinning disc confocal microscopes with lasers 488 and 561 with 10s time intervals.

Supplemental video 3

RPE1 CAV1-eGFP lysine-less mutant cells stably expressing RAB8a-mCherry. Time lapse live cell images recorded of cells serum-starved in phenol-free DMEM imaged with spinning disc confocal microscopes with lasers 488 and 561 with 10s time intervals.
